# MTHFD2 is required for DNA repair and implicated in LUAD radiotherapy resistance

**DOI:** 10.1186/s12967-026-07680-7

**Published:** 2026-01-09

**Authors:** Qian Huang, Weiwei Ouyang, Shengfa Su, Zhu Ma, Yinxiang Hu, Yichao Geng, Xiaxia Chen, Qingsong Li, Wengang Yang, Bing Lu

**Affiliations:** 1 Department of Thoracic Oncology, Affiliated Hospital of Guizhou Medical University, Guiyang, 550000 China; 2 Department of Thoracic Oncology, Affiliated Cancer Hospital of Guizhou Medical University, Guiyang, China; 3 Teaching and Research Department of Oncology, Clinical Medical College of Guizhou Medical University, Guiyang, China

**Keywords:** LUAD, MTHFD2, Radiation resistance, DNA damage repair, XRCC6

## Abstract

**Background:**

DNA damage-based radiotherapy (RT) is a conventional and effective local treatment for lung adenocarcinoma (LUAD). However, residual or recurrent tumors often occur due to radio-resistance. The underlying mechanisms by which radio-resistance occurs are still not fully understood. Novel predictive markers and potential therapeutic targets for LUAD radio-resistance need to be investigated.

**Methods:**

Bioinformatic tools were used to evaluate the biological functions of MTHFD2 in LUAD patients. The association of MTHFD2 with radiotherapy efficacy in LUAD patients was confirmed by immunohistochemistry. The baseline radioresponsiveness of different LUAD cells was identified and cells with acquired radio-resistance were generated. MTHFD2 in malignant phenotype and DNA damage after irradiation (IR) in LUAD cells were investigated by Western blotting, colony formation and neutral comet assays, etc. LC-MS and co-immunoprecipitation analyses were used to determine the interaction between MTHFD2 and XRCC6. Mouse models were used to verify these effects in vivo.

**Results:**

In this study, we showed that methylenetetrahydrofolate dehydrogenase 2 (MTHFD2) plays an important role in intrinsic and acquired radio-resistance in LUAD. High MTHFD2 expression correlates with poor RT response and reduced radiation-induced DNA damage. Mechanistically, MTHFD2 directly interacts with XRCC6 to facilitate XRCC6/XRCC5 complex formation, promoting non-homologous end joining (NHEJ)-mediated repair of DNA double-strand breaks, as evidenced by LC-MS and co-immunoprecipitation (Co-IP).

**Conclusions:**

This study establishes MTHFD2 as a master regulator of NHEJ-mediated radio-resistance through direct interaction with XRCC6. Our findings propose MTHFD2 expression as both a predictive biomarker for radiotherapy stratification and a therapeutic target for radiosensitization in LUAD.

**Supplementary information:**

The online version contains supplementary material available at 10.1186/s12967-026-07680-7.

## Background

Lung cancer remains the most common malignancy worldwide and is the leading cause of cancer-related mortality according to GLOBOCAN 2022 estimates [1]. As the predominant histological subtype of non-small cell lung cancer, lung adenocarcinoma (LUAD) accounts for approximately 50% of all diagnosed cases [2].

Current therapeutic paradigms for LUAD, including surgical resection [3], platinum-based chemotherapy [4], precision radiotherapy(RT) [5], targeted therapies against driver mutations [6], and immune checkpoint blockade [7] have significantly improved clinical management. Nevertheless, more than 60% of LUAD patients present with advanced disease at initial diagnosis [8] and frequently development of acquired resistance to RT regimens. RT remains a cornerstone treatment modality for all stages of LUAD, with 76.8% of non-small cell lung cancer patients receiving RT during their disease course from the SEERStat database [9]. Unfortunately, the efficacy of RT is disparate in LUAD patients is variable due to tumor cell radio-resistance [10].

RT induces DNA damage by both direct and indirect mechanisms: directly by causing lethal DNA lesions in irradiated cells, and indirectly by generating reactive oxygen species that target rapidly proliferating cells [11]. The spectrum of radiation-induced DNA damage includes base mutations, single-strand breaks (SSBs), and double-strand breaks (DSBs), with DSBs being the most critical lesion type [12]. Cellular repair pathways address DSBs through two main mechanisms: non-homologous end joining (NHEJ) and homologous recombination (HR).NHEJ is an error-prone repair mechanism that rapidly rejoins broken DNA ends with minimal homology requirements, while promoting cell cycle redistribution to establish radiation-resistant cellular states [13, 14]. In contrast, HR operates as a high-fidelity repair mechanism dependent on homologous chromatid templates during late S/G2 phases [15]. Failure to achieve timely and accurate DNA damage repair can lead to in mutagenic chromosomal rearrangements and subsequently drive the evolution of tumor cell radio-resistance through compromised radiation responsiveness [16].

Tumor cells develop intrinsic radio-resistance through genomic and/or epigenetic alterations, whereas radiotherapy-induced selective pressure mediates acquired resistance predominantly through genomic reprogramming during radiation exposure [17, 18].

Methylenetetrahydrofolate dehydrogenase 2 (MTHFD2) is a mitochondrial one-carbon metabolic enzyme that catalyzes the conversion of methylenetetrahydrofolate (CH2-THF) to formyltetrahydrofolate (CHO-THF) using NADP+ as the hydrogen acceptor, accompanied by the generation of NADPH [19]. In addition to its canonical metabolic role, accumulating evidence highlights the non-metabolic functions of MTHFD2 in oncogenesis. Clinical analyses consistently associate MTHFD2 overexpression with poor prognosis in various malignancies, including bladder cancer [20], breast cancer [21], non-small cell lung cancer [22], hepatocellular carcinoma [23], renal cell carcinoma [24], etc. Mechanistically, MTHFD2 promotes tumor cell migration and invasion [25, 26], and is also involved in DNA repair processes. Experimental models show that its nuclear localization facilitates HR repair through EXO1 interaction in mouse embryonic stem cells [27], and also enhances NHEJ-mediated DNA repair via PARP3 binding in tumor cells [28].

Our study shows for the first time that MTHFD2 critically regulates both intrinsic and acquired radio-resistance in LUAD cells. Clinically, high MTHFD2 expression leads to radio-resistance in patients. Functional validation confirmed that MTHFD2 depletion significantly increases radiation-induced DSBs and enhances radiosensitivity in LUAD models. Mechanistically, co-immunoprecipitation (Co-IP) analysis revealed that MTHFD2 interacts with XRCC6 to stabilize the KU70/KU80 complex, which is essential for NHEJ and subsequently facilitates the recruitment of the LIG4/XRCC4/XLF repair complex to DNA damage sites. Our study suggests that MTHFD2 is a promising therapeutic target for improving RT outcomes in LUAD.

## Methods

### Patients and tissue samples

Patients with primary LUAD diagnosed by pathological examination at the Affiliated Cancer Hospital of Guizhou Medical University from January 2020 to December 2023 were included. The inclusion criteria were as follows: 1) radical RT combined with paclitaxel and platinum-based chemotherapy for the primary site; 2) before and during RT and chemotherapy, the primary tumor was not treated with radical surgical resection, targeted, immune and other anti-tumor therapy; 3) no primary tumors in other sites; 4) complete personal basic information, RT dose, target area, imaging data and pathological data. The immunohistochemical method was used to analyse the paraffin section samples of the patients before treatment. Treatment response was assessed according to the RECIST v1.1 criteria for the evaluation of response in solid tumors. All patients signed an informed consent form. This study was approved by the Ethics Review Committee of the Affiliated Cancer Hospital of Guizhou Medical University.

### Bioinformatic analysis

The MTHFD2 expression profile of LUAD cases was retrieved from The Cancer Genome Atlas (TCGA) database. Tumor samples were stratified into high and low MTHFD2 expression cohorts based on median expression levels. Bioinformatic analyses were performed using an R-based workflow incorporating the following packages “DESeq2” for differential expression analysis. GSEA and KEGG pathway analyses were systematically performed using the “dplyr” package for data manipulation. Enriched pathways meeting pre-defined statistical thresholds (|NES| > 1, *p*value < 0.05 and FDR q-value < 0.25) were identified as statistically significant in accordance with GSEA methodological guidelines.

### Cell culture

Human LUAD cell lines (PC-9, H1975, H1299, A549) and the normal lung cell line Beas-2B were purchased from Pricella Life Science &Technology Co.,Ltd (Wuhan, China) and were validated by mycoplasma testing and short tandem repeat profiling analyses. All cell lines were cultured in RPMI-1640 medium or DMEM medium (Gibco, USA) supplemented with 10% FBS and 1% penicillin/streptomycin. All cells above were maintained at 37 °C and 5% CO2 in a humidified incubator.

### Establishment of radioresistant cell lines

PC-9 and H1299 cells were exposed to 2 Gy of homogenous X-ray using a biological irradiator (3 mm copper filter; 160 kV; 25 mA; source-to-skin distance (SSD): 50 cm; dose rate: 1.25 Gy/min; Rad Source Technologies, RS2000pro-225, USA). When the cells had undergone repair and repopulation and reached a density of 70–80%, a new X-ray irradiation cycle could be performed. Each cell line received a total dose of 60 Gy of X-rays (2 Gy per fraction × 5 fractions per week). The cells then passaged for more than 3 generations, and once the cells were growing stably, they were frozen and stored. The radioresistant cell lines were named PC9RR and H1299RR.

### Clonogenic survival assay

Different numbers of cells (400,800,1600/well) were seeded in 6-well plates and exposed to indicated single dose of irradiation (0, 2 and 4 Gy). After culturing for 10 to 14 days of growth, the colonies were fixed with paraformaldehyde and stained with crystal violet. The number of colonies was then calculated and analysed using image J software. Colonies with > 50 cells were counted. The ratio of counted colonies to inoculated cells was normalized to the plating efficiency at 0 Gy under the same conditions for each experiment.

PE (Plating efficiency) % = (Colonies/Input cells) ×100

SF (Surviving fraction) % = PE/PE(0 Gy) ×100

### Cell counting Kit-8 (CCK8) assay

For the CCK8 assay, cells (2 × 10^3^ cells/well) were seeded in 96-well plates and exposed to 6 Gy of X-rays. 10 μL CCK8 reagent (APExBIO, USA) was added to each well and incubated for 2 h in the dark to measure cell viability at different time points, respectively. Absorbance values were measured at 450 nm using a microplate reader (BioTek, USA).

### Comet assay

The comet assay was performed using reagents from the DNA Damage Detection Kit (KeyGEN BioTECH, China). In summary, cells were mixed with low-melting agarose gel at the indicated proportion and spread onto comet slides. The slides were then lysed in ice lysis buffer, followed by electrophoresis in alkaline electrophoretic buffer. Following this, the gels were neutralized with a Tris–HCl buffer on three occasions, each time for 10 min. The gels were then stained with PI and photographed using a fluorescent microscope. The quantitation of tail moments was analysed by CASP Software, and at least 50 cells were measured in each group.

### Western blotting (WB)

The cells were lysed in RIPA buffer containing protease inhibitors (Epizyme Biotech, China) and then subjected to centrifugation in order to collect the supernatant. Protein concentrations were then measured using a BCA assay (Solarbio, China). The samples were then heated in a 100 °C metal bath for 10 min. Nuclear and cytoplasmic protein extractions were conducted using a Nuclear and Cytoplasmic Protein Extraction Kit (Beyotime, China), according to the manufacturer’s instructions. Proteins were separated via SDS-PAGE and electrophoretically transferred to a PVDF membrane (Millipore, USA). The membrane was blocked with 5% non-fat milk or BSA in TBST for 1 h at room temperature. The membrane was then incubated overnight at 4 °C with primary antibody dilution buffer. Following three washes with TBST, the membrane was probed with an HRP-conjugated secondary antibody for one h at room temperature. After three additional washes, protein signals were detected using an ECL substrate (Yeasen, China) and visualized via chemiluminescence imaging (Clinx, China). Primary antibodies were listed in Table S1.

### Transfection and construction of stable overexpression and knockdown cell lines

Recombinant lentiviral vectors carrying MTHFD2/XRCC6 overexpression constructs (LV-MTHFD2/LV-XRCC6) and short hairpin RNA (shRNA) sequences targeting MTHFD2/XRCC6 (LV-shMTHFD2/LV-shXRCC6) were commercially purchased from GeneChem Co., Ltd (Shanghai, China). Viral particles were mixed with HitransG P infection enhancer (GeneChem, China) at optimized multiplicity of infection (MOI) ratios and incubated with cells for 16 h. After transduction, the viral supernatant was replaced with fresh complete medium supplemented with puromycin (Beyotime, China) for 7 days. The shRNA and siRNA target sequences (validated by Sanger sequencing) are listed in Supplementary Table S2 and S3. Parallel negative controls included empty vector-transfected cells and non-targeting shRNA treatments.

### Immunohistochemistry (IHC)

Formalin-fixed paraffin-embedded tissue sections were sequentially deparaffinized through a graded xylene series followed by rehydration in an ethanol gradient. Antigen retrieval was performed with sodium citrate buffer. After endogenous peroxidase quenching with 3% H₂O₂, sections were blocked with 5% normal goat serum (Solarbio, China). Primary antibody incubation was performed for 16 h at 4 °C in a humidified chamber. Visualization was achieved using HRP-conjugated secondary antibodies with DAB chromogen (Solarbio, China) under microscopic control. Nuclei were counterstained with Mayer’s haematoxylin (Solarbio, China), followed by differentiation in 0.5% acetic acid alcohol and blue staining in 0.02% ammonia water. Whole slide imaging was performed using the Vectra Polaris automated quantitative pathology system (PerkinElmer). Protein expression was scored using a validated histoscore (H-score) system combining staining intensity and cellular distribution: Intensity grading: 0 (negative), 1+ (weak), 2+ (moderate), 3+ (strong); percentage stratification: 1: ≤25%, 2: 26–50%, 3: 51–75%, 4: > 75%. The composite score (intensity × percentage) was calculated for each sample. Samples with cumulative scores ≥6 were considered to have high expression, whereas scores < 6 indicated low expression. Primary antibodies were listed in Table S1.

### Mass spectrometry

Direct binding partners of MTHFD2 were isolated by Co-IP experiments under physiological buffer conditions. Precipitated protein complexes were resolved by SDS-PAGE and visualised by colloidal Coomassie Brilliant Blue G-250 staining. Distinct protein bands exceeding background staining intensity were excised for in-gel processing. Gel slices were sequentially destained, reduced and alkylated. Trypsin digestion followed by peptide extraction. Desalting was performed using C18 ZipTips (Millipore, USA) prior to LC-MS/MS analysis. Chromatographic separation was performed using a nanoElute UHPLC system (Bruker) coupled to a timsTOF Pro 2 mass spectrometer (Bruker) via a CaptiveSpray source. Peptides were loaded onto a 25 cm Aurora Series column using a 90 min gradient. MS acquisition was performed in parallel accumulation serial fragmentation mode with 10 MS/MS events per cycle. The mass spectra database retrieval software used in this project was MaxQuant 2.0.1.0 with a 1% FDR threshold at both peptide spectrum match and protein level.

### Immunofluorescence staining

Cells were fixed with 4% paraformaldehyde for 20 min at room temperature. Cell samples were then sequentially processed: membrane permeabilization with 0.3% Triton X-100 (Solarbio, China) for 10 min and blocking with 5% normal goat serum for 1 h. Samples were then incubated with primary antibodies overnight at 4 °C, followed by appropriate fluorophore-conjugated secondary antibodies for 1 h. Nuclear counterstaining was performed with DAPI (Beyotime，China) for 5 min. Fluorescence images were captured using an inverted epifluorescence microscope and quantitative analysis was performed using ImageJ software.

### Co-immunoprecipitation (Co -IP)

Cells were lysed using immunoprecipitation lysis buffer (Elabscience, China) supplemented with phosphatase/protease inhibitor cocktail. 40 μL protein A/G agarose beads (Elabscience, China) were conjugated with 2 μg of target-specific antibodies by incubation for 1 h at room temperature (22–25°C) with gentle rotation. The antibody-bound beads were then incubated with 500 μg of protein lysate at 4 °C for 12–16 h with constant agitation to facilitate antigen-antibody complex formation. After magnetic bead precipitation, the supernatant was removed with a magnetic frame. The bead complexes were then washed three times with ice-cold PBS and resuspended in ice-cold PBS and loading buffer (Epizyme Biotech, China). Protein denaturation was performed by heating the samples at 100 °C for 5 min using a dry bath incubator. Finally, the eluted proteins were separated by SDS-PAGE and analysed by Western blotting using standard protocols.

### Xenograft tumor model

Male BALB/c nude mice (5–7 weeks old) were purchased from Vital River Laboratories (Beijing, China). All mice were provided with food and water ad libitum and maintained in dedicated pathogen-free facilities under a 12 h light/dark cycle. For the mouse tumor xenograft assays, mice were injected subcutaneously with 3 × 10^6^ H1299 cells. For the radiosensitivity experiment, mice were treated with a dose of 2 Gy for 5 consecutive days when the xenografts had an average volume of 150 mm^3^. After 28 days of maintenance, the mice were sacrificed and the tumors were dissected. The tumor volume (mm^3^) was calculated using the formula: V = 0.5 × length × width^2^, and the dissected subcutaneous tumor was weighed. All procedures followed the ARRIVE guidelines and were approved by the Ethics Committee of Guizhou Medical University (Protocol No. 210,203).

### Flow cytometry assay

Apoptotic progression in LUAD cells was quantitatively assessed using a dual fluorescence Annexin V-APC/7-AAD apoptosis detection kit (KeyGEN BioTECH, China). Briefly, adherent cells were enzymatically dissociated with 0.25% EDTA-free trypsin (Gibco, USA), washed twice in ice-cold phosphate-buffered saline and pelleted by centrifugation. Cell pellets were then resuspended in 500 μL of pre-chilled Annexin V binding buffer at a density of 1 × 10^6^ cells/mL. After sequential staining with 5 μL Annexin V-APC and 5 μL 7-amino-actinomycin D under light-protected conditions, cell fluorescence was immediately analysed using a flow cytometer (Becton, USA).

## Statistical

All quantitative results are presented as mean ± SEM of at least three biological replicates. The log-rank test was used for K-M survival analysis. A two-tailed unpaired Student t test was used to compare variables between two groups, and one-way ANOVA was used for multigroup comparisons. Differential expression levels of MTHFD2 were analysed by Wilcoxon rank sum test. The correlation of MTHFD2 expression with clinical features was analysed by Kruskal-Wails rank sum test. The significance threshold was set at *p* < 0.05. All statistical analyses were performed using GraphPad 9.5.1 software (USA) and R 4.3.2 software.

## Results

### Discovery of MTHFD2 as a radioresistant oncogene in LUAD

**Fig. 1:**
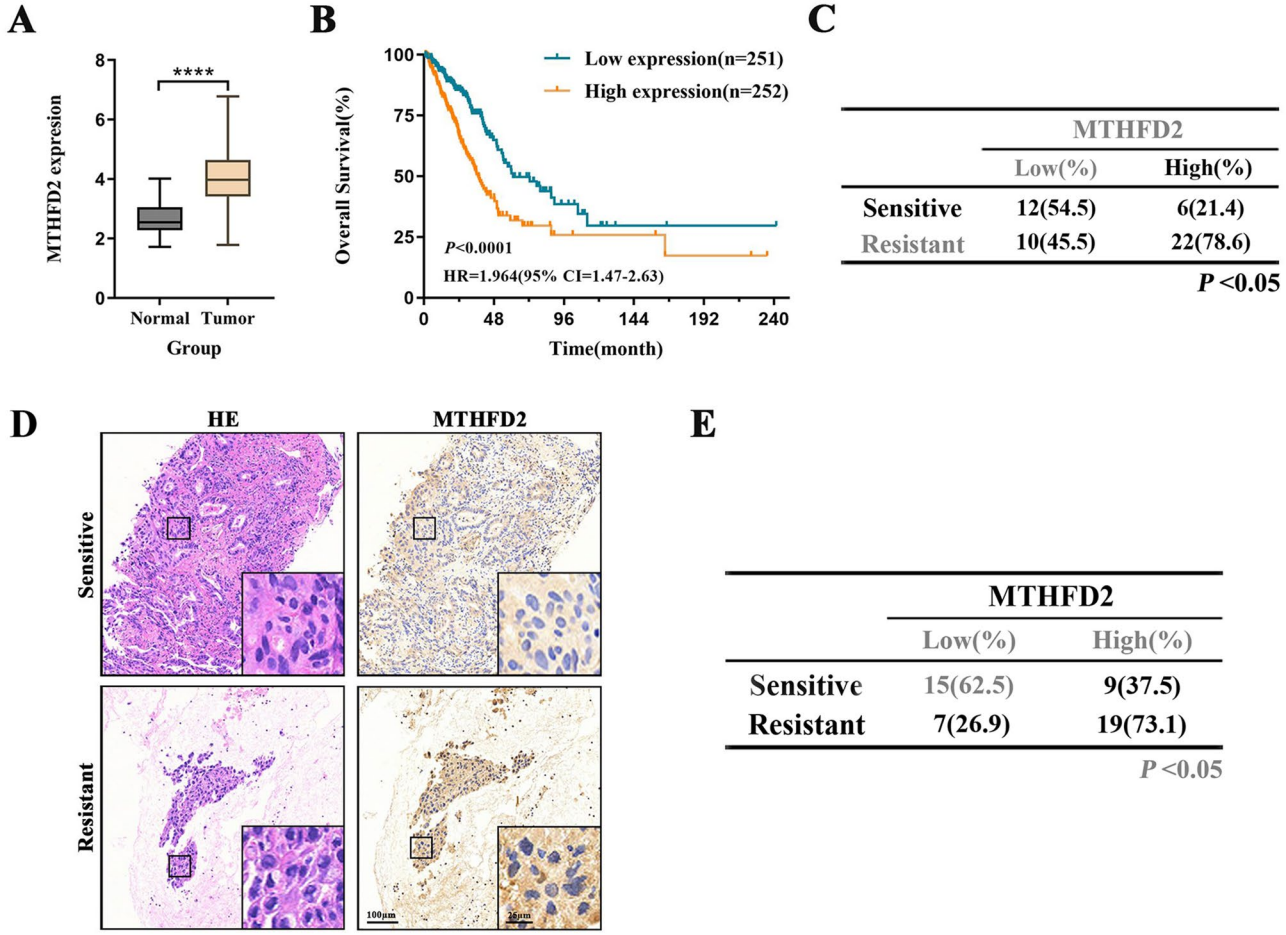
Discovery of MTHFD2 as a radioresistant oncogene in LUAD. **A** Comparative analysis of MTHFD2 expression between LUAD tumours (*n* = 503) and adjacent normal lung tissues (*n* = 59) in TCGA datasets (****p < 0.0001). **B** Kaplan-meier survival curves demonstrating significantly reduced overall survival (OS) in radiotherapy-treated LUAD patients with high MTHFD2 expression. **C** Radiogenomic stratification showing elevated MTHFD2 levels in radiotherapy-resistant tumours. **D** Representative histological sections: Hematoxylin-eosin (HE) staining and immunohistochemical (IHC) localization of MTHFD2 in treatment-sensitive versus resistant LUAD cases. Scale bars: 100 μm/25 μm(inset). **E** Quantitative analysis of MTHFD2 IHC H-scores stratified by chemoradiotherapy response

To investigate the expression profile of MTHFD2 in LUAD and its association with chemoradiotherapy response, we found that MTHFD2 mRNA expression was upregulated in LUAD tissue (*n* = 503) compared to that in normal tissue (*n* = 59) (Fig. 1A) in TCGA database. Stratification of radiotherapy-treated LUAD patients by median MTHFD2 expression demonstrated that high-expression patients exhibited significantly reduced overall survival (Fig. 1B), with RT response analysis confirming elevated MTHFD2 levels in radiotherapy-resistant tumors (Fig. 1C). To validate these findings, we performed immunohistochemical (IHC) analysis on pretreatment biopsies from 50 LUAD patients undergoing chemoradiotherapy (baseline characteristics in Table 1). Quantitative IHC scoring revealed significantly lower MTHFD2 expression in treatment-sensitive patients compared to resistant cases (Figs. 1D and 1E). Notably, while no baseline differences in gender, smoking history, or TNM staging were observed between groups, patients with metastatic disease demonstrated higher chemoradiotherapy resistance. These findings establish MTHFD2 overexpression as a characteristic feature of LUAD, with its expression levels demonstrating significant inverse correlations with both patient survival and therapeutic response to chemoradiation.

**Table 1:** Baseline characteristics of 50 LUAD patients according to the chemoradiotherapy response

Futures	Group	Chemoradiotherapy Response		*P*** value**
		Sensitive	Resistant	
Age(y)	≤60	12	14	0.7827
	＞60	10	14	
Gender	Female	5	3	0.277
	Male	17	25	
Smoking status	Never Smoking	9	8	0.3857
	Smoking	13	20	
T Stage	T1-2	6	10	0.5589
	T3-4	16	18	
N stage	N2	10	13	0.1532
	N3	12	15	
M stage	M0	12	6	0.0202*
	M1	10	22	

### RT induces LUAD cell radio-resistance by evoking MTHFD2 overexpression

**Fig. 2:**
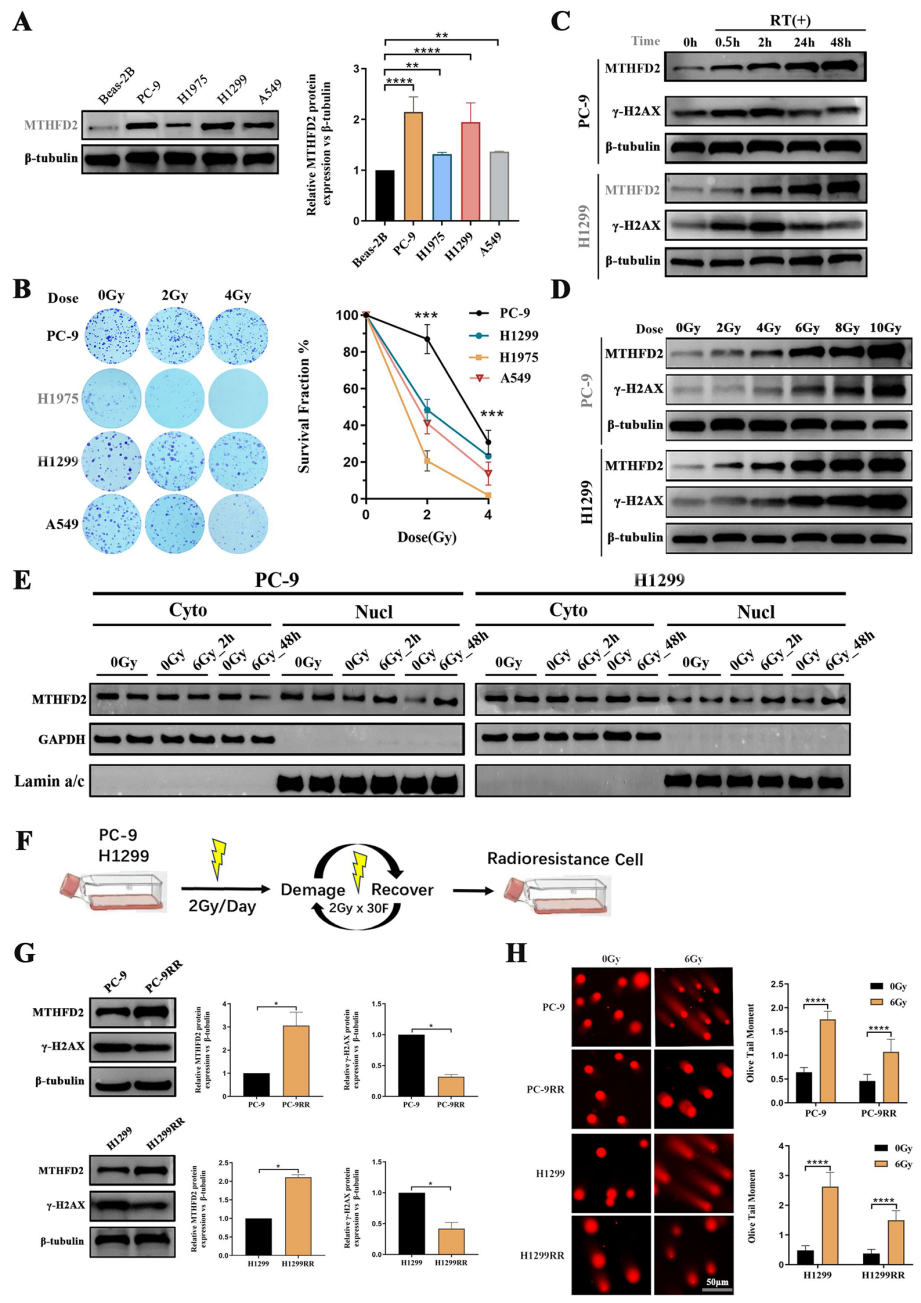
RT induces LUAD cell radio-resistance by evoking MTHFD2 overexpression. **A** Baseline MTHFD2 expression: left: immunoblots of MTHFD2 protein levels across LUAD cell lines. Right: densitometric quantification normalized to β-tubulin. **B** Left: Representative colony formation images post-X-ray irradiation (0–4 Gy). Right: cell survival curves. (**C**-**D**) Radiation-induced MTHFD2 regulation: (**C**) time-course immunoblot (0–48 h post-6 Gy) demonstrating temporal coordination of MTHFD2 and γ-H2AX expression. (**D**) dose-dependent upregulation (0–10 Gy) of MTHFD2 and DNA damage markers. **E** Nuclear/cytoplasmic fractionation showing radiation-triggered (6 Gy) MTHFD2 nuclear accumulation over time (lamin a/c nuclear marker, GAPDH cytoplasmic control). **F** Schematic of iterative fractionated irradiation (2 Gy × 30 fractions) for generating radioresistant clones. **G** Left: immunoblots of MTHFD2 and γ-H2AX in parental vs. radioresistant lines. Middle and right: quantitative comparison showing MTHFD2 elevation and γ-H2AX reduction in resistant clones. **H** DNA repair capacity assessment: left: neutral comet assay images post-6 Gy irradiation. Right: olive tail moment quantification demonstrating impaired DNA repair in radioresistant cells (scale bar: 50 μm). Data represent mean ± SD from three biologically independent experiments. Statistical significance determined by two-tailed Student’s t-test (*n* = 3, *p < 0.05, **p<0.001，***p<0.001, ****p < 0.0001)

First, we compared protein expression between a normal human bronchopulmonary epithelial cell line (Beas-2B) and human LUAD epithelial cell lines (PC9, H1975, H1299, and A549). As shown in Fig. 2A, MTHFD2 showed significantly higher expression levels in four LUAD epithelial cell lines compared to Beas-2B. In particular, H1299 and PC9 cells showed the most pronounced upregulation, which led to their selection for subsequent gene silencing experiments. Furthermore, given the heterogeneity in radiation sensitivity between different cell lines, we performed colony formation assays to identify radiation-resistant candidates for downstream analysis. Four LUAD epithelial cell lines were exposed to single dose X-ray irradiation. As shown in Fig. 2B, the survival curves revealed distinct radiobiological responses among the cell lines. Significantly, PC-9 and H1299 cells showed significantly preserved clonogenic capacity compared to H1975 and A549 at equivalent radiation dose, leading to their selection for subsequent experiments.

Given the established role of ionizing radiation in inducing DNA damage responses, we exposed LUAD cell lines to RT and quantified γ-H2AX (a well-known biomarker of DNA double-strand breaks) and MTHFD2 protein dynamics at sequential post-irradiation intervals. Time-course analysis revealed coordinated yet distinct expression patterns: MTHFD2 showed sustained up-regulation (0–48 h post-RT), whereas γ-H2AX showed transient induction followed by resolution (Fig. 2C). Notably, MTHFD2 expression showed a radiation dose-dependent increase, which was highly consistent with the expression trend of γ-H2AX (Fig. 2D). Furthermore, we observed its progressive nuclear accumulation in a time-dependent manner (Fig. 2E). These findings mechanistically link MTHFD2 to DNA damage repair processes, consistent with our initial hypothesis.

Then we established radiation-resistant LUAD cell lines (PC-9, H1299) (Fig. 2F). During cumulative radiation exposure, cells underwent sequential DNA damage-repair cycles, with surviving cells (PC-9RR, H1299RR) demonstrating acquired radio-resistance. Phenotypic validation using colony formation assays and CCK-8 assays confirmed enhanced proliferation (Figures S1A-B) and attenuated radiation-induced DNA damage (Fig. 2G-H) in radioresistant lines compared to parental controls. Concurrently, radioresistant cells exhibited progressive upregulation of MTHFD2 protein expression (Fig. 2G), demonstrating radiation-inducible regulation of MTHFD2 during acquired resistance development.

### MTHFD2 silencing cooperates with RT to exacerbate DNA damage and enhance potentiate antitumor efficacy

**Fig. 3:**
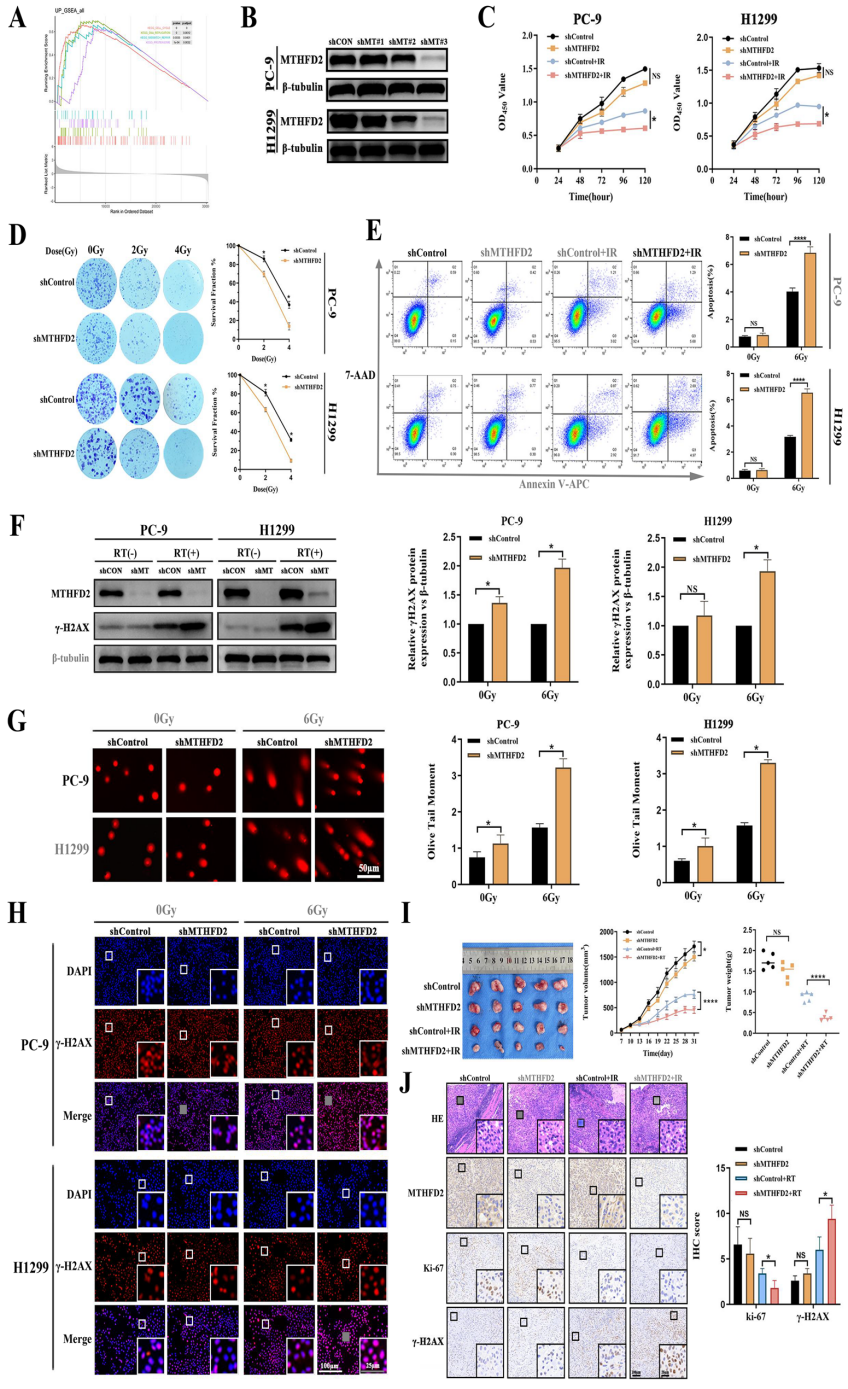
MTHFD2 silencing cooperates with RT to exacerbate DNA damage and enhance antitumour efficacy. **A** GSEA enrichment plot highlighting DNA damage repair pathways in MTHFD2-high tumours. **B** Representative immunoblots of MTHFD2 protein levels in PC-9 and H1299 cells transduced with shRNA constructs. (**C**-**E**) shRNA-mediated MTHFD2 depletion significantly enhanced RT sensitivity (6 Gy) as evidenced by: (**C**) impaired proliferation (CCK-8 assay, 24–120 h post-RT), (**D**) Reduced clonogenic survival (14-day colony formation). (**E**) Increased apoptosis (flow cytometry, Annexin V/7-AAD staining at 48 h). (**F**-**H**) DNA damage potentiation: (**F**) Immunoblot showing γ-H2AX upregulation (left: representative blot; right: quantification relative to β-tubulin, (**G**) Comet assays demonstrating elevated DNA fragmentation (left: representative images; right: tail moment quantification) (scale bar: 50 μm), (**H**) Immunofluorescence microscopy showing γ-H2AX upregulation. Scale bars: 100 μm/25 μm(inset). **I** Schematic of xenograft irradiation protocol (left). Tumor growth kinetics (middle: volume; right: endpoint weight) in nude mice bearing H1299-shMTHFD2 or shControl xenografts ± fractionated RT (2 Gy × 5 fractions). Data expressed as mean ± SEM (*n* = 5/group). **J** Tumor biomarker analysis: Representative IHC staining showing reduced MTHFD2/Ki67 and increased γ-H2AX in irradiated shMTHFD2 tumours versus controls. Scale bars: 100 μm/25 μm(inset). Data represent mean ± SD from three biologically independent experiments. Statistical significance determined by two-tailed Student’s t-test (*n* = 3, *p < 0.05, **p < 0.01, ****p < 0.0001, ns: not significant)

To further investigated the underlying mechanisms, GSEA pathway analyses of TCGA data revealed divergent pathway activation patterns between different MTHFD2 expression groups (Fig. 3A). MTHFD2 high-expression group showed enrichment in DNA replication, HR, and cell cycle regulation, suggesting enhanced process of cell replication and DNA damage repair. Conversely, MTHFD2 low-expression group preferentially activated glycerophospholipid metabolism and arachidonic acid pathways (Table S4).

Based on the radiation-inducible expression profile of MTHFD2 in LUAD cells, we employed shRNA-mediated knockdown combined with RT to investigate its functional role in tumor radio-resistance. Western blot analysis confirmed efficient MTHFD2 suppression in PC-9 and H1299 cells (Fig. 3B, Figure S2). Functional characterization revealed that MTHFD2 deficiency significantly attenuated cellular proliferation (Fig. 3C) and clonogenic survival (Fig. 3D), with enhanced radiosensitivity further evidenced by flow cytometry (apoptosis induction) (Fig. 3E). Furthermore, MTHFD2 silencing exacerbated radiation-induced DNA damage, as demonstrated by elevated γ-H2AX expression via western blot (Fig. 3F), increased comet tail moments (Fig. 3G) and the amplified γ-H2AX foci formation (Fig. 3H, Figure S3). These findings collectively indicate a compromised DNA repair capacity in MTHFD2-deficient cells, and this disparity between the two groups was further exacerbated following 6Gy treatment. In vivo validation using xenograft models demonstrated a synergistic suppression of tumor growth when MTHFD2 knockdown was combined irradiation (Figure S4, Fig. 3I). Immunohistochemical analysis of resected tumors revealed dual effects including reduced proliferative index (Ki-67+ cell depletion) and sustained DNA damage (γ-H2AX+ cell accumulation) in the combination therapy group versus radiation monotherapy (Fig. 3J). These findings establish that MTHFD2 overexpression in LUAD correlates with adverse clinical outcomes and treatment resistance, potentially mediated through DNA damage repair pathway activation.

### MTHFD2 interacts with XRCC6 and controls XRCC6 expression

**Fig. 4:**
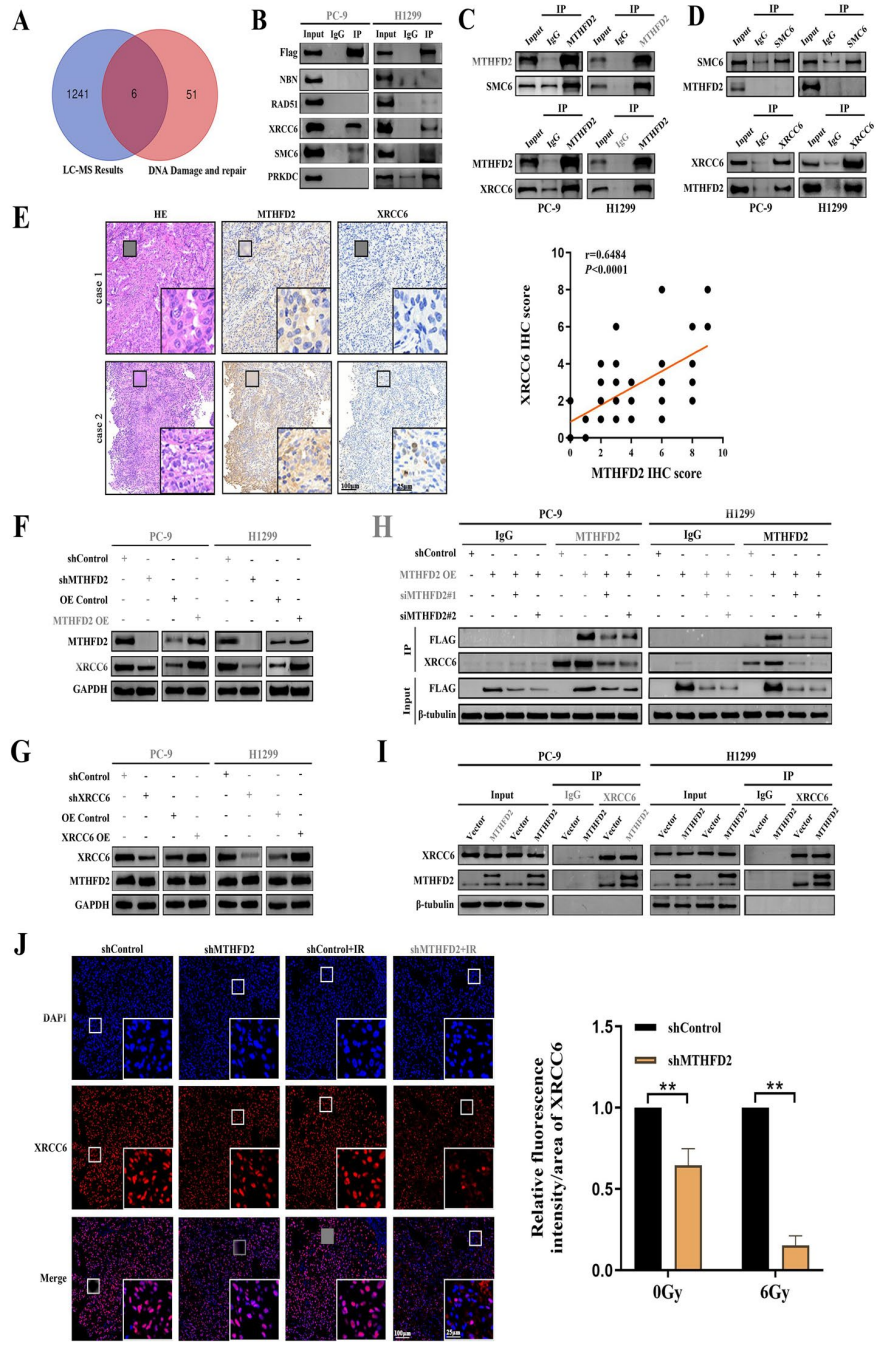
MTHFD2 interacts with XRCC6 and controls XRCC6 expression. **A** Venn diagram illustrating the overlap between MTHFD2-associated proteins identified by LC-MS/MS analysis (*n* = 1,247) and curated DNA repair factors (HR/NHEJ pathways). Six candidate interactors are highlighted. **B** Co-IP validation of MTHFD2-binding partners in H1299 cells using anti-flag antibody. **C** Co-immunoprecipitation assay detection of MTHFD2, SMC6 and XRCC6 with endogenous MTHFD2 in whole-cell lysates. **D** Co-immunoprecipitation assay detection of MTHFD2, SMC6 and XRCC6 with endogenous SMC6 and XRCC6 in whole-cell lysates. **E** Correlation between MTHFD2 and XRCC6 expression levels in radiotherapy-sensitive (case 1) versus radiotherapy-resistant (case 2) LUAD cohorts. Scale bars: 100 μm/25 μm(inset). **F** XRCC6 protein levels were positively regulated in cells with MTHFD2 deletion or overexpression. **G** XRCC6 perturbation (knockdown or overexpression) failed to alter MTHFD2 protein abundance. **H** Co-IP analysis of FLAG-MTHFD2/XRCC6 interaction dynamics in MTHFD2-manipulated LUAD cells (β-tubulin as loading control). **I** Co-IP confirming interaction specificity: XRCC6 antibody pulls down exogenous MTHFD2 in overexpression models. **J** in vivo functional correlation: MTHFD2 depletion (shRNA) impairs XRCC6 expression in xenograft tumours post-ionizing radiation treatment, as shown by dual immunofluorescence test. Scale bars: 100 μm/25 μm(inset). Statistical significance determined by two-tailed Student’s t-test (*n* = 3, **p < 0.01)

To explore the mechanism of MTHFD2 in DNA damage repair, we performed immunoprecipitation assays using H1299 cell lysates transfected with FLAG-MTHFD2. Immunoprecipitation with anti-FLAG antibodies was followed by LC-MS/MS analysis. This analysis identified 1247 candidate proteins potentially associated with MTHFD2 (Table S5). Subsequent intersection analysis with a curated database of DNA damage repair proteins (HR and NHEJ pathway) revealed six overlapping interactors: SMC6, RAD51, NBN (NBS1), XRCC6, XRCC5, and PRKDC (Table S6; Fig. 4A and B). Notably, exogenous Co-IP assays in lung cancer cells confirmed a selective interaction between MTHFD2 and XRCC6 (Ku70) or SMC6, but not with other candidates (Fig. 4B). Furthermore, endogenous Co-IP revealed only XRCC6 interacts with MTHFD2 (Fig. 4C and D). In LUAD patient tumor tissues, MTHFD2 and XRCC6 expression exhibited significant positive correlation (Spearman *r* = 0.6484, *p* < 0.0001, Fig. 4E). The XRCC6 (Ku70)/XRCC5 (Ku80) heterodimer is essential for NHEJ-mediated DNA repair. In subsequent experiments, we quantified heterodimer levels by measuring XRCC6 expression. To elucidate the functional relationship between MTHFD2 and XRCC6, we modulated MTHFD2 expression by shRNA-mediated knockdown or overexpression. Intriguingly, XRCC6 protein levels exhibited direct correlation with MTHFD2 expression (Fig. 4F), whereas reciprocal manipulation of XRCC6 failed to alter MTHFD2 abundance (Fig. 4G). Immunofluorescence co-localization assays further corroborated this interaction in xenograft tumor tissues (Fig. 4J). Subsequent Co-IP experiments using lung cell lysates with either MTHFD2 overexpression or silencing demonstrated dynamic modulation of the MTHFD2–XRCC6 interaction (Fig. 4H and I). Collectively, these findings establish that MTHFD2 directly binds to XRCC6 and regulates its protein stability under physiological conditions.

### XRCC6 participates in MTHFD2-mediated DNA NHEJ repair

**Fig. 5:**
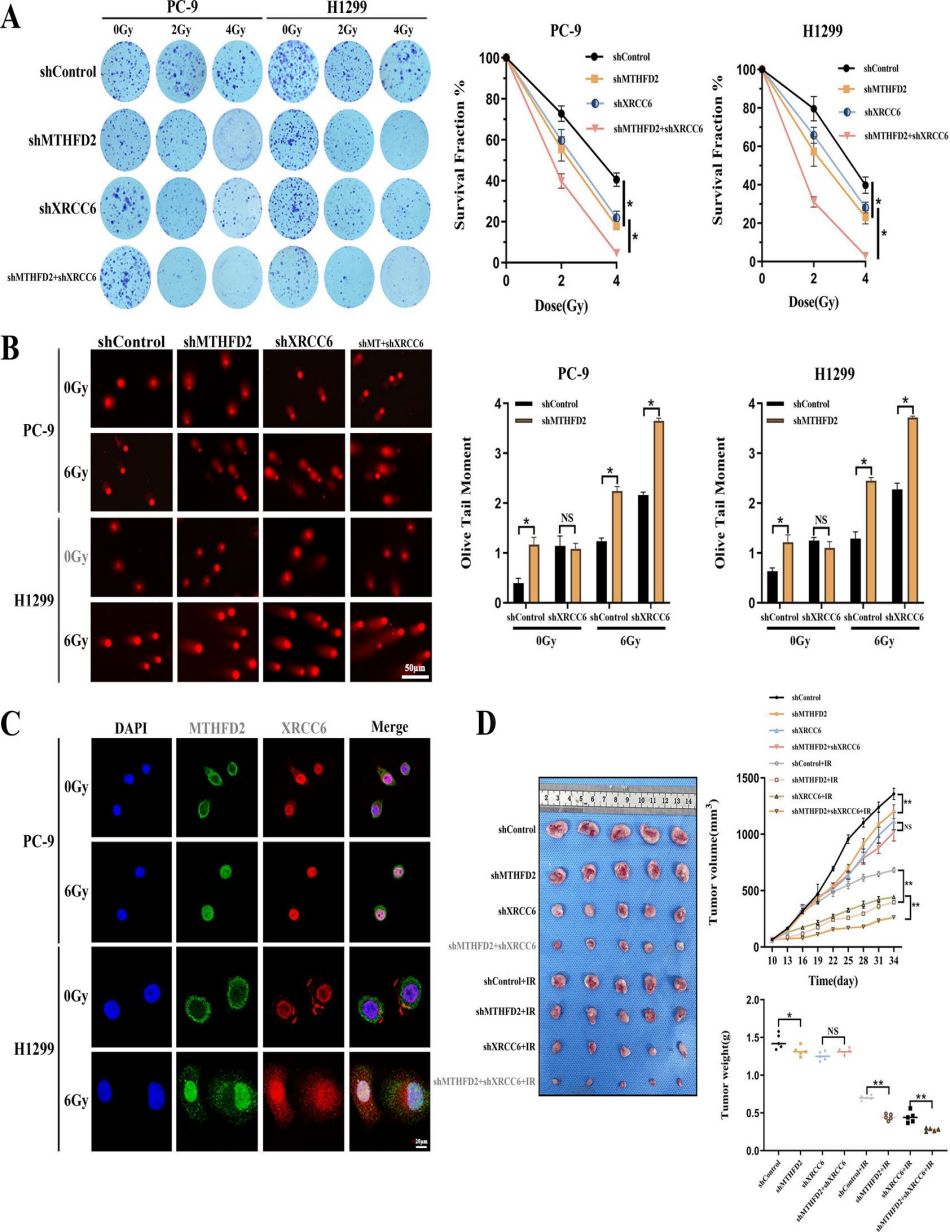
MTHFD2 participates in XRCC6-mediated DNA NHEJ repair. **A** RT sensitivity assessment: left - representative colony formation images; right - quantified survival curves. **B** DNA damage quantification: left - neutral comet assay images post-RT; right - olive tail moment analysis (Scale bar: 50 μm). **C** Radiation-induced nuclear co-localization: immunofluorescence staining of MTHFD2 (green) and XRCC6 (red) with DAPI counterstaining (blue) (scale bar: 20 μm). **D** in vivo RT response: left - representative tumours images; middle - tumor growth curves; right - terminal tumours weights (*n* = 5/group). Data represent mean ± SD from three biologically independent experiments. Statistical significance determined by two-tailed Student’s t-test (*n* = 3, *p < 0.05, **p < 0.01, ns: not significant)

XRCC6 has been identified as a critical mediator for NHEJ DNA repair pathway. Our findings demonstrate that MTHFD2 silencing significantly reduces XRCC6 protein levels under basal cellular conditions. To explore whether MTHFD2 performs its DNA damage repair functions in a XRCC6 dependent manner, we generated isogenic LUAD cell models with single or dual depletion of MTHFD2 and XRCC6 via shRNA. Notably, XRCC6 silencing further reduced the colony-forming capacity in MTHFD2-deficient cells following radiation therapy (Fig. 5A), implying that MTHFD2 promotes proliferation of LUAD cells in an XRCC6-dependent manner. Parallel DNA damage quantification through neutral comet assays revealed that individual depletion of either protein significantly increased IR-induced DNA strand breaks, and combined depletion further exacerbated the damage (Fig. 5B). In addition, subcellular co-localization analysis demonstrated radiation-enhanced nuclear interaction between MTHFD2 and XRCC6 (Fig. 5C). These results indicate that MTHFD2 not only regulates XRCC6 expression but also is responsible for the function of DNA damage repair. In vivo validation using subcutaneous xenografts showed that single-agent depletion of MTHFD2 or XRCC6 synergized with RT versus without RT, whereas dual depletion provided additive benefit (Fig. 5D). Altogether, these data establish that MTHFD2 exerts its DNA repair functions through direct regulation of XRCC6 within the NHEJ pathway.

### XRCC6 overexpression partially rescues MTHFD2 knockdown-induced functional impairment

**Fig. 6:**
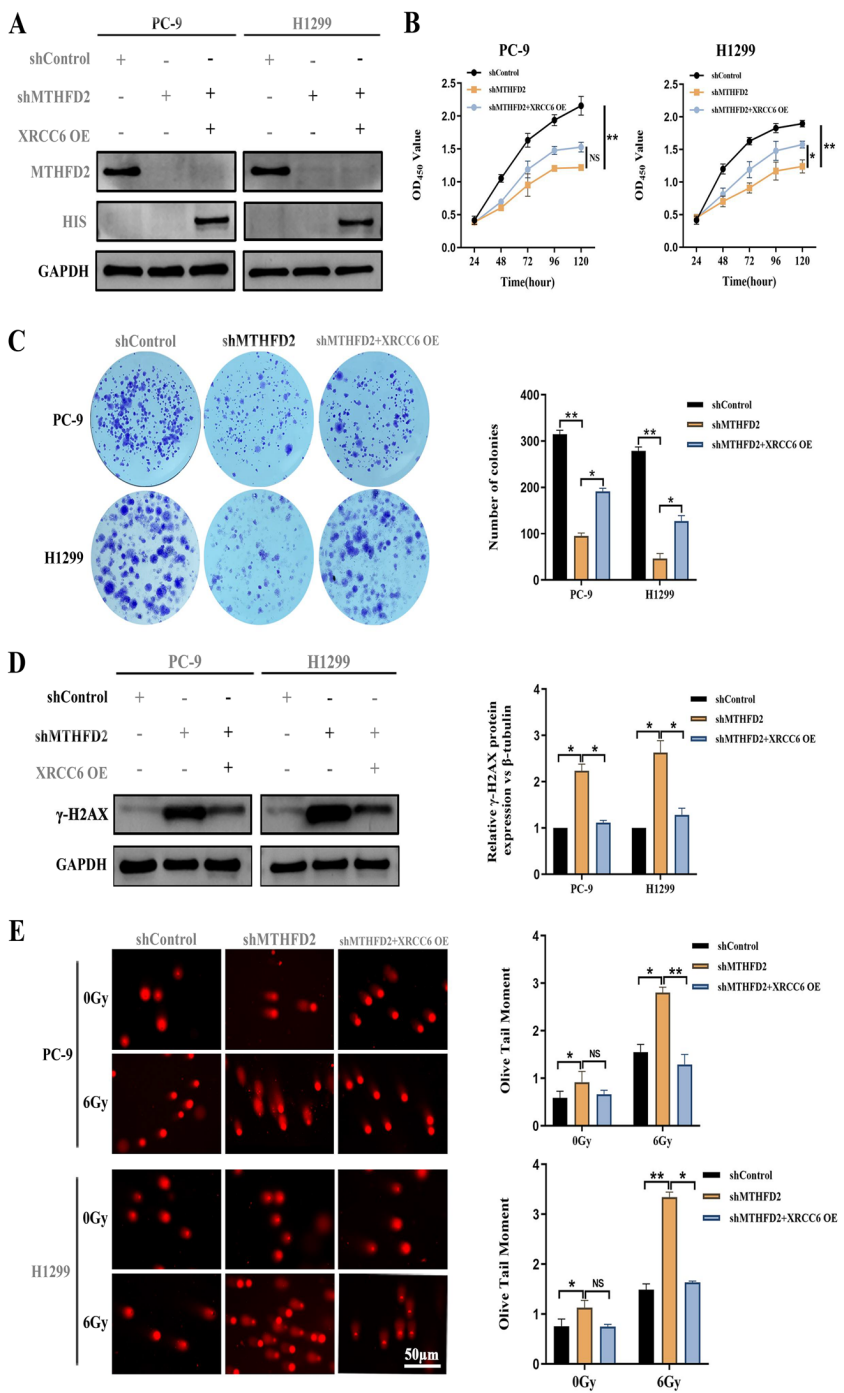
XRCC6 overexpression partially rescues MTHFD2 knockdown-induced functional impairment. **A** Immunoblot analysis of MTHFD2 and XRCC6 expression in LUAD cells transfected with specific shRNA or overexpression constructs. **B** Cell viability rescue: Cell Counting Kit-8 (CCK-8) assays demonstrated that XRCC6 overexpression restored cellular viability in MTHFD2-silenced cells. **C** Clonogenic recovery: quantitative colony formation assays confirmed partial reversal of proliferation defects by XRCC6 reconstitution. **D** DNA damage mitigation: γ-H2AX immunoblotting post-6 Gy irradiation showed XRCC6 overexpression reduced DNA damage signaling. **E** Comet assay validation: neutral comet quantification revealed XRCC6 reconstitution shortened olive tail moments in MTHFD2-deficient cells (scale bar: 50 μm). Data represent mean ± SD from three biologically independent experiments. Statistical significance determined by two-tailed Student’s t-test (*n* = 3, *p < 0.05, **p < 0.01)

Our findings have demonstrated that MTHFD2 was involved in XRCC6-mediated DNA repair. To investigate the functional hierarchy between these proteins, we developed a rescue system by overexpressing XRCC6 in MTHFD2-silenced LUAD cells after radiotherapy. CCK-8 proliferation kinetics showed a time-dependent attenuation of growth suppression upon XRCC6 overexpression (Fig. 6B). Consistent with this observation, quantitative clonogenic assays revealed that XRCC6 reconstitution partially restored the colony-forming capacity in MTHFD2-depleted cells with RT (Fig. 6C). Radiation challenge experiments (6 Gy) coupled with γ-H2AX immunoblotting confirmed functional complementarity: MTHFD2 knockdown elevated DNA damage signaling, which was substantially mitigated by XRCC6 overexpression (Fig. 6D). Quantification of neutral comet assay further corroborated this rescue effect, showing that XRCC6 reconstitution reduced Olive tail moments in MTHFD2-deficient cells (Fig. 6E). Collectively, these findings demonstrate that XRCC6 overexpression compensates for MTHFD2 loss by restoring DNA repair fidelity, thereby reestablishing radio-resistance in LUAD models.

### MTHFD2 recruits the LIG4/XRCC4/XLF complex to activate DNA damage repair pathways

**Fig. 7:**
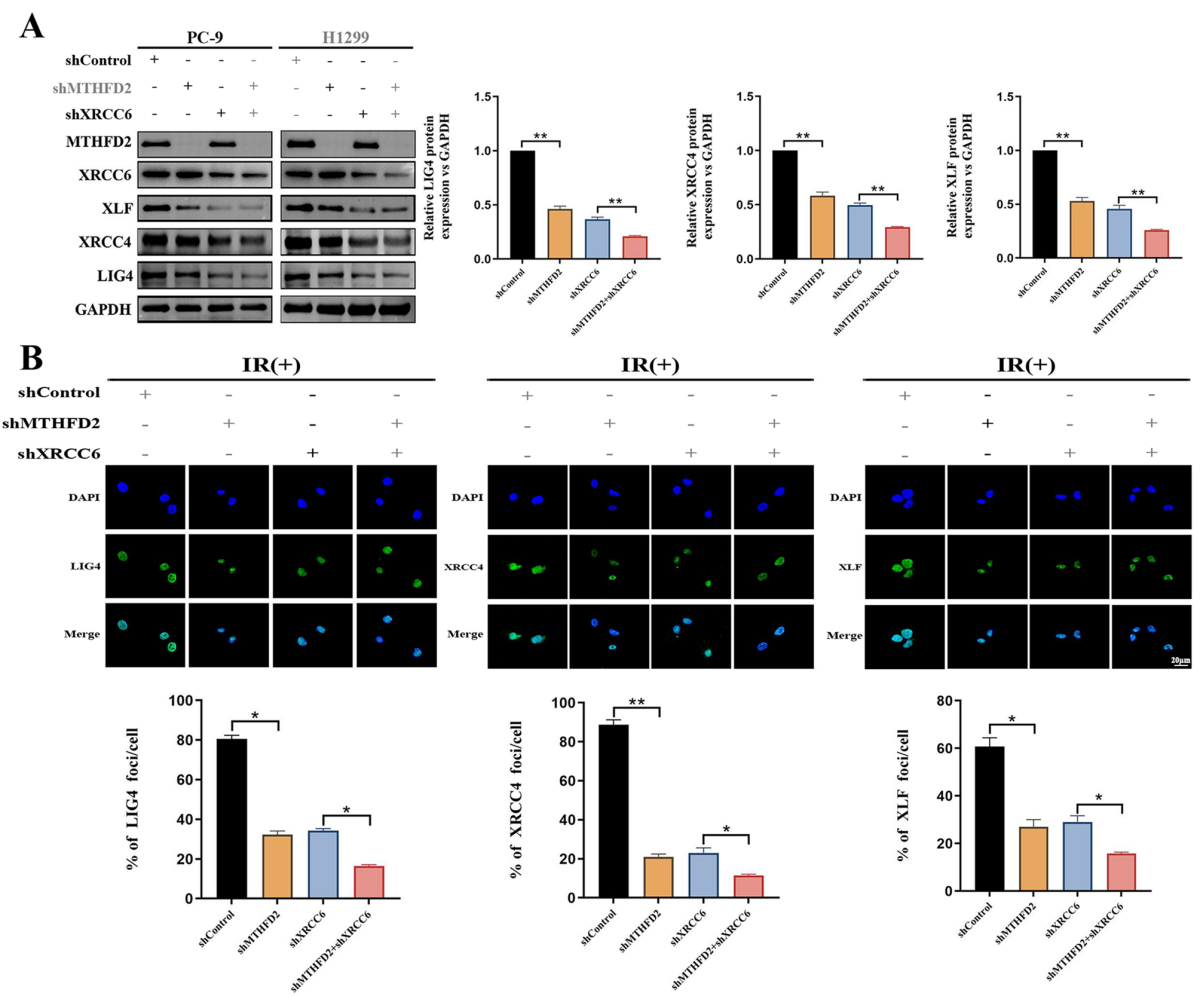
MTHFD2 recruits the LIG4/XRCC4/XLF complex to activate DNA damage repair pathways. **A** Immunoblot analysis of key NHEJ components (MTHFD2, XRCC6, XLF, XRCC4, LIG4) in LUAD cells transfected with specific shRNA. **B** Immunofluorescence revealed that MTHFD2 depletion markedly attenuated nuclear foci formation of LIG4/XRCC4/XLF complexes (Scale bar: 20 μm). Data represent mean ± SD from three biologically independent experiments. Statistical significance determined by two-tailed Student’s t-test (*n* = 3, *p < 0.05, **p < 0.01)

XRCC6 (Ku70) plays an essential role for assembling the LIG4/XRCC4/XLF complex at DSBs. Given the established MTHFD2–XRCC6 interaction and regulatory axis, we postulated that MTHFD2 could orchestrate NHEJ efficiency through XRCC6-mediated recruitment of this repair complex. As demonstrated in Fig. 7A–B, ionizing radiation-induced nuclear accumulation of LIG4/XRCC4/XLF was markedly attenuate upon MTHFD2 depletion. Crucially, the complex was further reduced in cells that knocked down MTHFD2 and XRCC6 at the same time. These findings delineate a sequential recruitment mechanism: MTHFD2 maintains XRCC6 protein homeostasis, which in turn facilitates the spatiotemporal assembly of the core NHEJ machinery at DSB sites. The XRCC6-dependent attenuation of downstream LIG4 complex formation under MTHFD2 depletion provides the mechanistic basis for the observed radiosensitization phenotype.

## Discussion

RT has become a cornerstone therapeutic modality for LUAD with technological innovations in precision radiation delivery [29]. However, heterogeneous clinical outcomes persist among patients, a phenomenon strongly associated with the development of radio-resistance [17, 18]. While significant research efforts have focused on identifying predictive biomarkers of radio-resistance in LUAD [30–32], no reliable clinically validated biomarkers have reached routine clinical implementation.

**Fig. 8:**
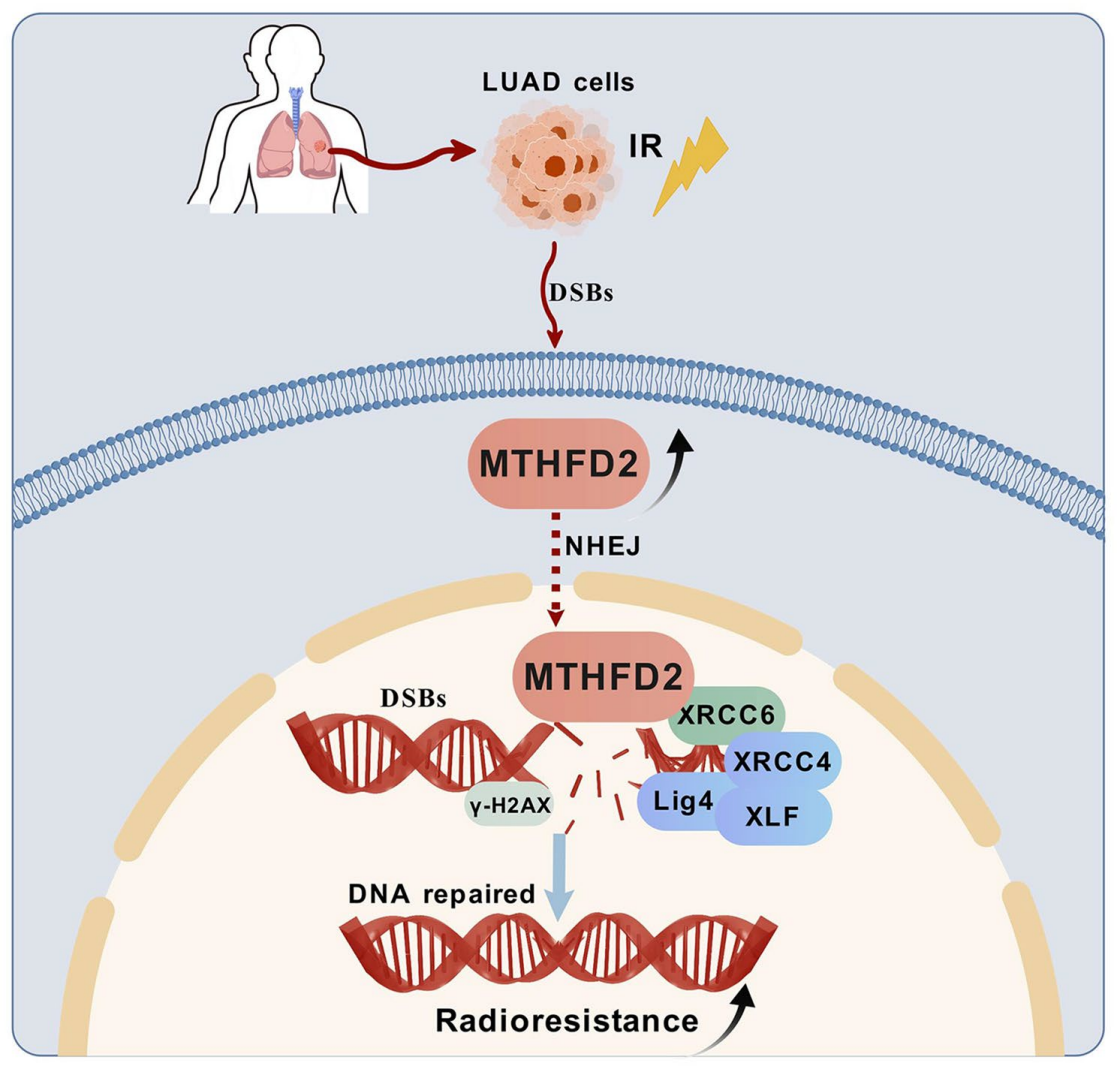
The schematic model for the possible mechanisms of MTHFD2-mediated radiation resistance of LUAD cells

This study uncovers a previously unrecognized role for MTHFD2 in conferring radio-resistance in LUAD. Systematic in vitro and in vivo studies show that elevated MTHFD2 expression correlates with radio-resistance phenotypes in both parental LUAD cells and radioresistant sublines, indicating its dual involvement in primary and acquired radio-resistance (Fig. 8). Mechanistically, MTHFD2 depletion exacerbated radiation-induced DSBs, as evidenced by increased γ-H2AX expression. Crucially, we found that MTHFD2 interacts with XRCC6 to NHEJ-mediated repair by recruiting the LIG4/XRCC4/XLF complex to sites of damage. These findings suggest targeting the MTHFD2/XRCC6 axis as a novel radiosensitization strategy for LUAD.

Consistent with previous reports implicating MTHFD2 in cancer cell proliferation and invasion [25, 33], our findings further establish its clinical relevance in radio-resistance. Immunohistochemical analysis revealed that patients with high MTHFD2 expression had a significantly poorer response to RT, providing the first clinical evidence linking MTHFD2 overexpression to intrinsic radio-resistance in LUAD. Notably, radioresistant sublines showed MTHFD2 upregulation associated with attenuated DNA damage accumulation, suggesting its involvement in acquired resistance via enhanced repair capacity. This phenomenon is consistent with previous evidence suggesting that radiation-induced genetic reprogramming may drive acquired radio-resistance through enhanced DNA repair capacity [17, 18, 34].

Our mechanistic studies show that MTHFD2 directly interacts with XRCC6 to orchestrate NHEJ repair. Co-IP assays confirmed physical binding between MTHFD2 and XRCC6, a core component of the XRCC6/XRCC5 heterodimer essential for NHEJ initiation [35–38]. Notably, MTHFD2 not only positively regulates XRCC6 expression but also enhances their interaction through radiation-induced nuclear translocation, significantly attenuating radiation-induced DNA DSBs. Interestingly, our rescue experiments revealed that overexpressing XRCC6 only partially reversed the radio-sensitization effects caused by MTHFD2 knockdown. This key observation suggests that while the interaction with and regulation of XRCC6 constitute a significant mechanism, it is likely not the sole pathway by which MTHFD2 promotes radio-resistance. Crucially, MTHFD2 facilitates the assembly of the LIG4/XRCC4/XLF complex via XRCC6 mediation, thereby completing the core NHEJ repair cascade [39–42]. While previous studies have established the role of the XRCC6/XRCC5 heterodimer in DSB end recognition [35–37, 43, 44], our work identifies MTHFD2 as a novel upstream regulator of this process. Importantly, despite the proteomic identification of multiple MTHFD2-interacting DNA repair proteins, functional validation revealed exclusive specificity for XRCC6, underscoring the biological relevance of this interaction.

While previous studies have predominantly characterized MTHFD2 through its canonical dehydrogenase activity in tumorigenesis [45, 46], our findings reveal a previously unrecognized role of this metabolic enzyme in DNA damage response pathways. This study provides the first experimental evidence linking MTHFD2 to the molecular machinery of genomic repair in LUAD, expanding its functional paradigm in cancer biology. Taken together with previous evidence that oncogenic KRAS directly upregulates MTHFD2 expression and that DNA damage response (DDR) pathways are critical for mediating resistance to KRAS inhibitors [45–48]. Given the established role of MTHFD2 in regulating cellular folate metabolism and nucleotide synthesis, which are essential for an efficient DDR, it is tantalizing to speculate that MTHFD2 may serve as a key functional effector linking oncogenic KRAS signaling to DDR activation and therapy resistance. We have framed this as a key future direction to elucidate the precise mechanistic underpinnings. Comprehensive analyses, such as metabolomic profiling and assessment of other DNA repair pathways like HR, will be essential to dissect these potential synergies.

This study has several limitations. First, although we have demonstrated that MTHFD2 modulates DNA damage repair through XRCC6, the precise mechanisms underlying MTHFD2 nuclear translocation and its interaction dynamics with XRCC6 (transcriptional, translational, or post-translational stability) remain uncharacterized and require further investigation. Second, the limited clinical sample size requires confirmation through multicenter cohort validation.

## Conclusions

In conclusion, this study systematically elucidates the mechanism by which MTHFD2 promotes radio-resistance in LUAD through regulation of the XRCC6-mediated NHEJ repair pathway. These findings not only provide novel insights into the metabolic-genomic interplay underlying tumor radio-resistance, but also provide a theoretical basis for the development of precision RT strategies based on MTHFD2 expression stratification.

## Electronic supplementary material

Below is the link to the electronic supplementary material.

Supplementary Material 1

Supplementary Material 2

Supplementary Material 3

Supplementary Material 4

Supplementary Material 5

Supplementary Material 6

Supplementary Material 7

Supplementary Material 8

Supplementary Material 9

Supplementary Material 10

Supplementary Material 11

Supplementary Material 12

## Data Availability

The datasets used and/or analyzed during the current study are available from the corresponding author on reasonable request.
