# Supplementary figures and images for: MTHFD2 is required for DNA repair and implicated in LUAD radiotherapy resistance
Source: J Transl Med. 2026 Jan 9;24:154. doi: 10.1186/s12967-026-07680-7 (PMC12882504; doi:10.1186/s12967-026-07680-7)

**A**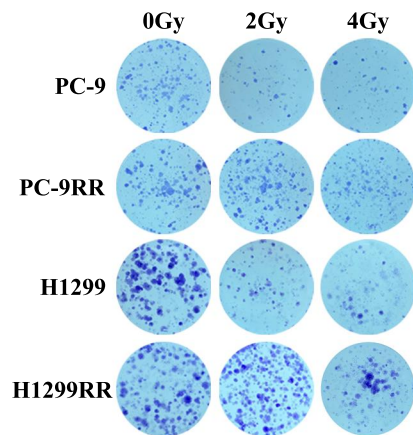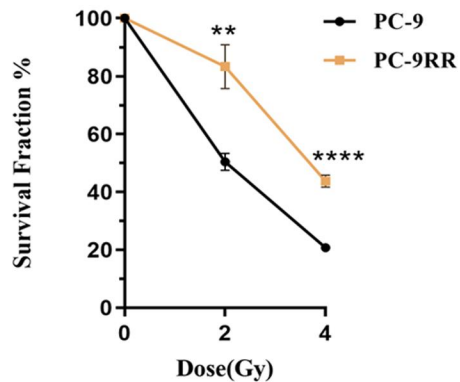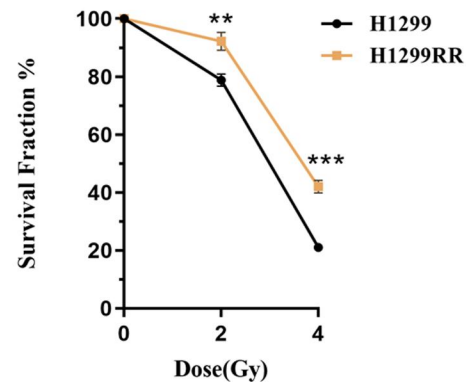**B**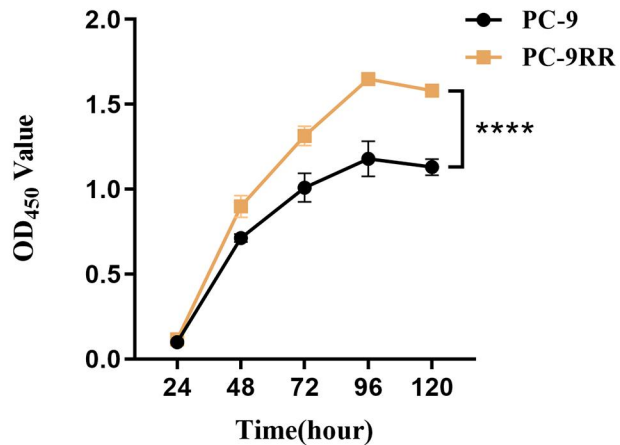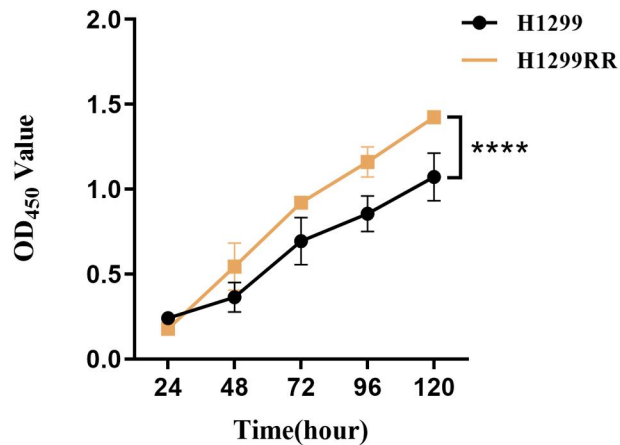

Supplement: Supplementary file 1 — Supplementary Material 1 [file 12967_2026_7680_MOESM1_ESM.pdf]

## PC-9

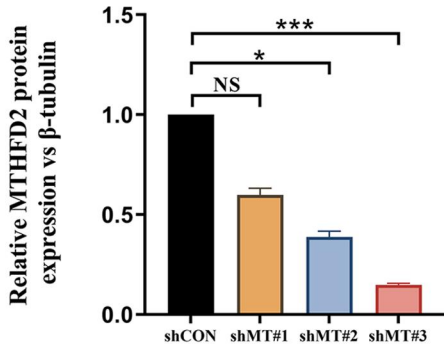

## H1299

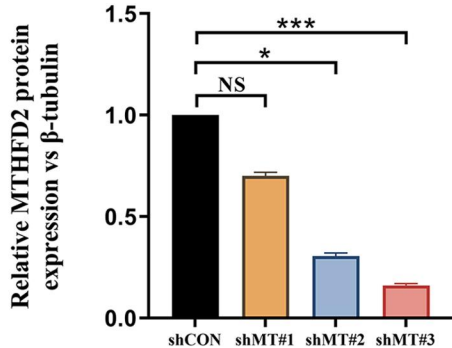

Supplement: Supplementary file 2 — Supplementary Material 2 [file 12967_2026_7680_MOESM2_ESM.pdf]

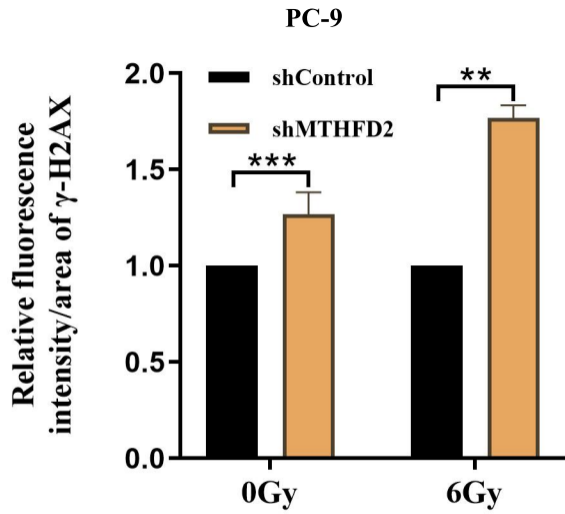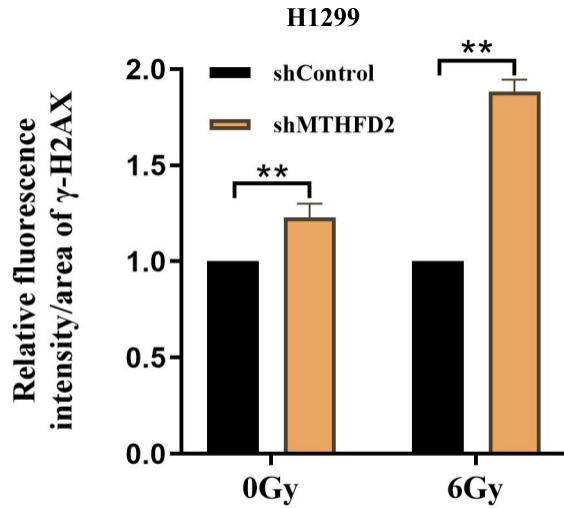

Supplement: Supplementary file 3 — Supplementary Material 3 [file 12967_2026_7680_MOESM3_ESM.pdf]

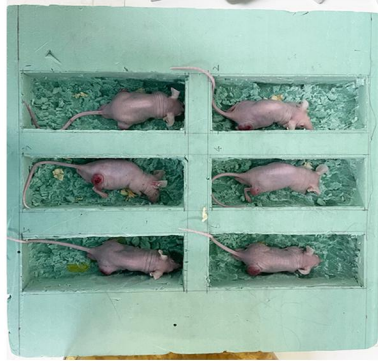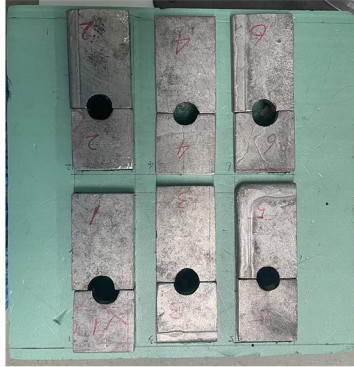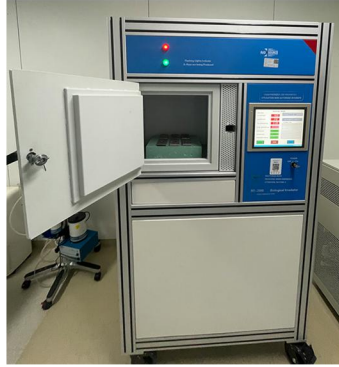

Supplement: Supplementary file 4 — Supplementary Material 4 [file 12967_2026_7680_MOESM4_ESM.pdf]
