# Supplementary material for: MTHFD2 is required for DNA repair and implicated in LUAD radiotherapy resistance
Source: J Transl Med. 2026 Jan 9;24:154. doi: 10.1186/s12967-026-07680-7 (PMC12882504; doi:10.1186/s12967-026-07680-7)
Supplement: Supplementary file 5 — Supplementary Material 5 [file 12967_2026_7680_MOESM5_ESM.docx]

**Supplementary Figure legends**

**Fig.S1(A-B) (A)** Clonogenic survival assay with dose-response curves comparing radioresistant clones and parental cells under gradient radiation (0-4 Gy) (Left: Representative crystal violet-stained colonies; Right: Normalized survival fraction. **(B)** CCK-8 proliferation kinetics showing enhanced viability in radioresistant clones post 6 Gy irradiation compared to parental controls. Data represent mean ± SD from three biologically independent experiments. Statistical significance determined by two-tailed Student's t-test (***P* < 0.01, ****P<*0.001, *****P* < 0.0001).

**Fig.S2** MTHFD2 knockdown validation: Quantification normalized to β-tubulin. Data represent mean ± SD from three biologically independent experiments. Statistical significance determined by two-tailed Student's t-test (**P* < 0.05, ****P<*0.001, ns: not significant).

**Fig.S3**Quantification of Immunofluorescence microscopy showing γ-H2AX upregulation. Data represent mean ± SD from three biologically independent experiments. Statistical significance determined by two-tailed Student's t-test (***P* < 0.01, ****P<*0.001).

**Fig.S4** Lead plate was used to cover the area outside the tumor body of nude mice, and the tumor body received RT model.
