# Supplementary material for: MTHFD2 is required for DNA repair and implicated in LUAD radiotherapy resistance
Source: J Transl Med. 2026 Jan 9;24:154. doi: 10.1186/s12967-026-07680-7 (PMC12882504; doi:10.1186/s12967-026-07680-7)
Supplement: Supplementary file 6 — Supplementary Material 6 [file 12967_2026_7680_MOESM6_ESM.pdf]

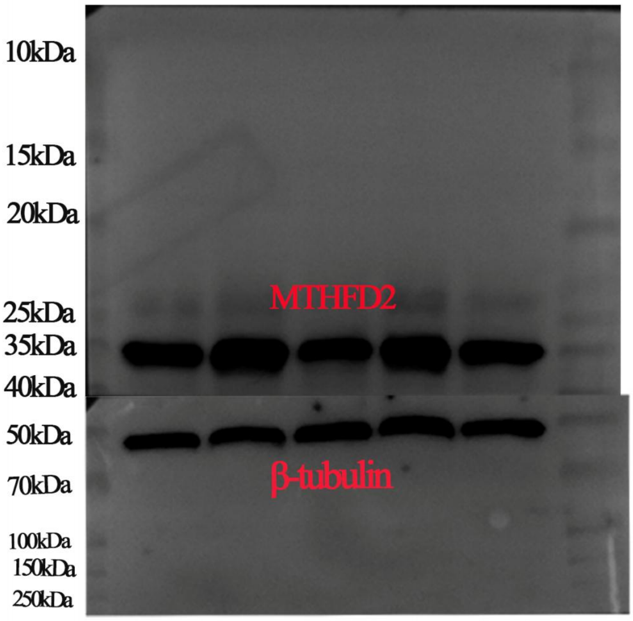
10kDa
15kDa
20kDa
25kDa
35kDa
40kDa
50kDa
70kDa
100kDa
150kDa
250kDa
MTHFD2
β-tubulin

Figure 2A

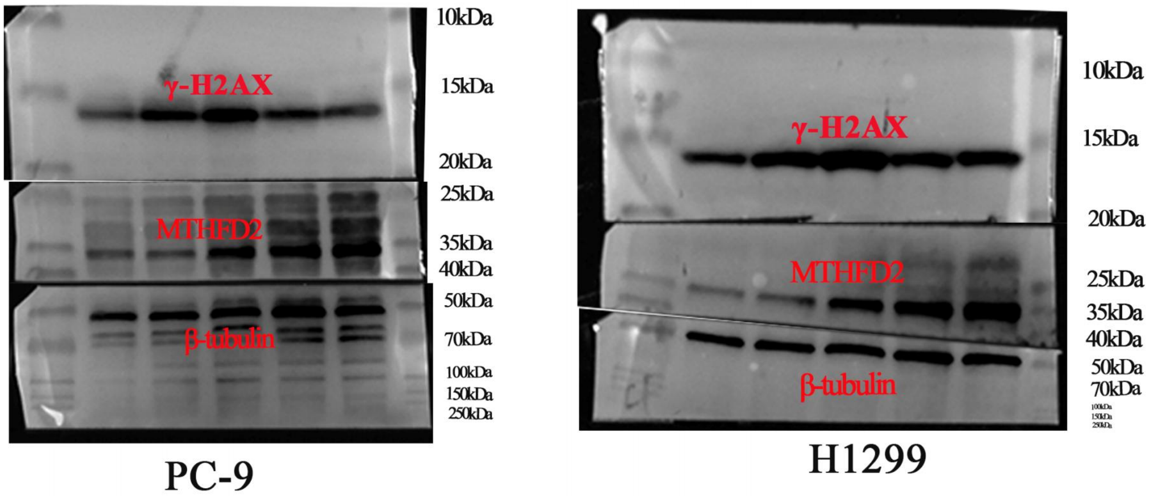

PC-9 H1299

Figure 2C

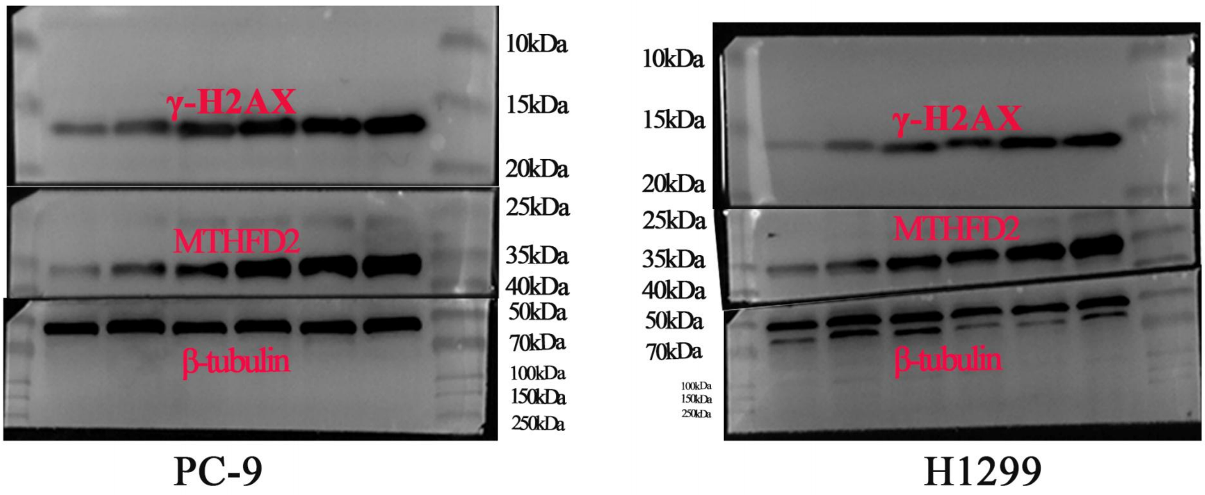
γ-H2AX
MTHFD2
β-tubulin
10kDa
15kDa
20kDa
25kDa
35kDa
40kDa
50kDa
70kDa
100kDa
150kDa
250kDa
PC-9
H1299

Figure 2D

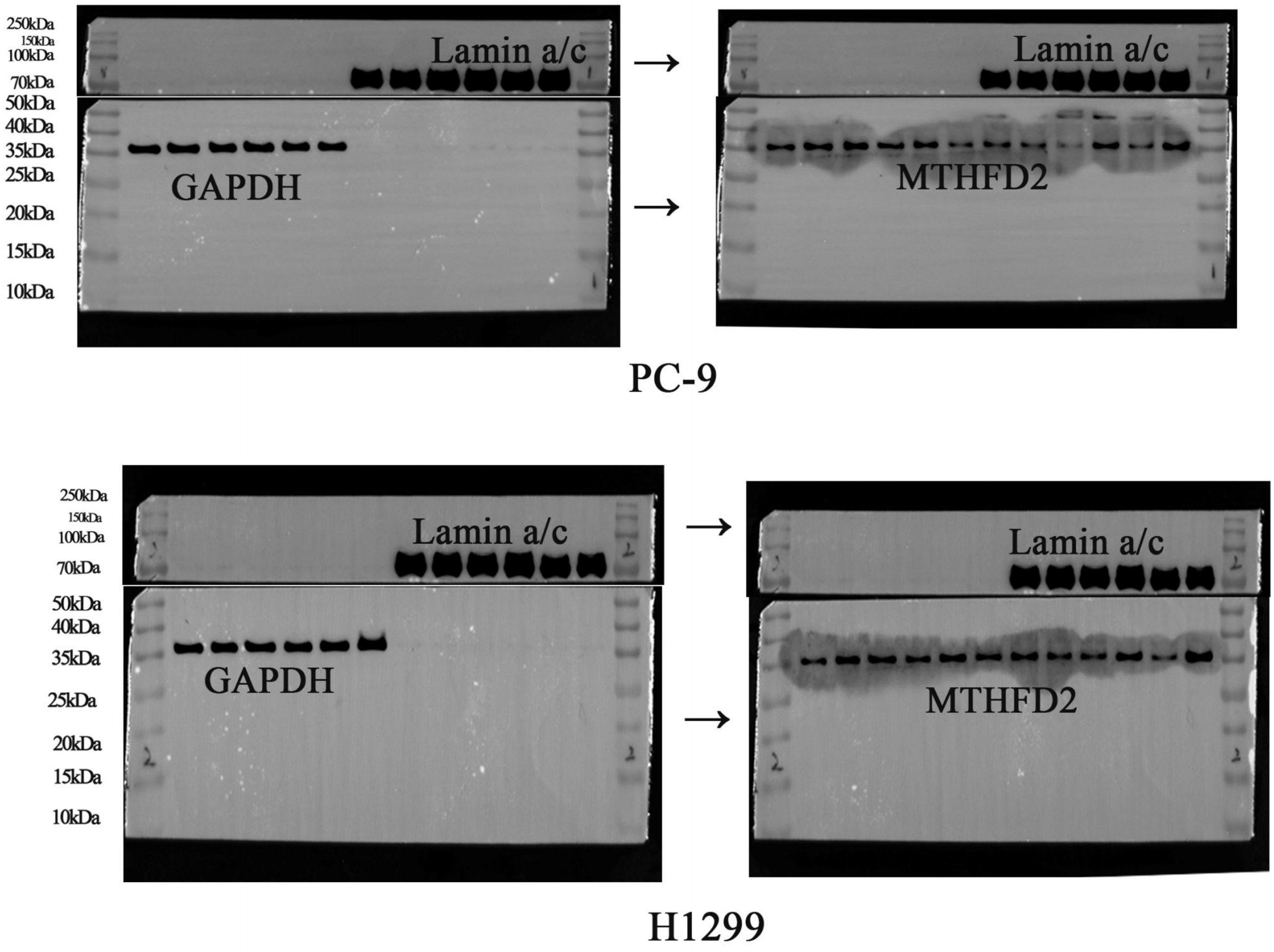

250kDa
150kDa
100kDa
70kDa
50kDa
40kDa
35kDa
25kDa
20kDa
15kDa
10kDa
Lamin a/c
GAPDH
MTHFD2
PC-9
H1299

Figure 2E

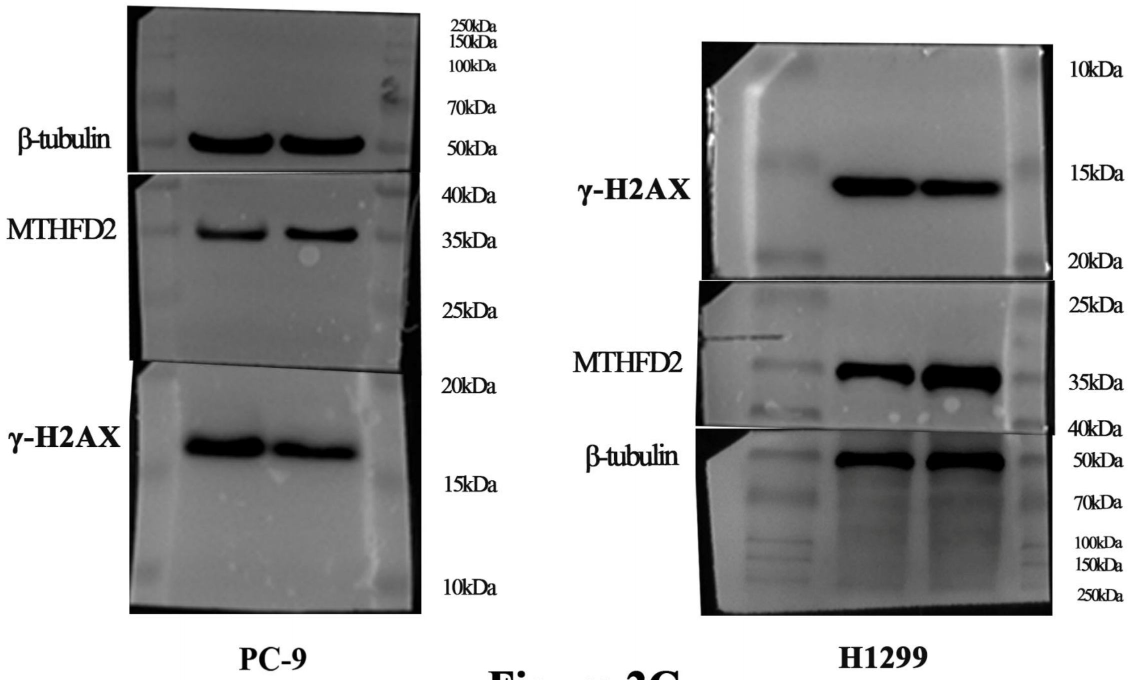
β-tubulin
MTHFD2
γ-H2AX
250kDa
150kDa
100kDa
70kDa
50kDa
40kDa
35kDa
25kDa
20kDa
15kDa
10kDa
PC-9
γ-H2AX
MTHFD2
β-tubulin
10kDa
15kDa
20kDa
25kDa
35kDa
40kDa
50kDa
70kDa
100kDa
150kDa
250kDa
H1299

Figure 2G

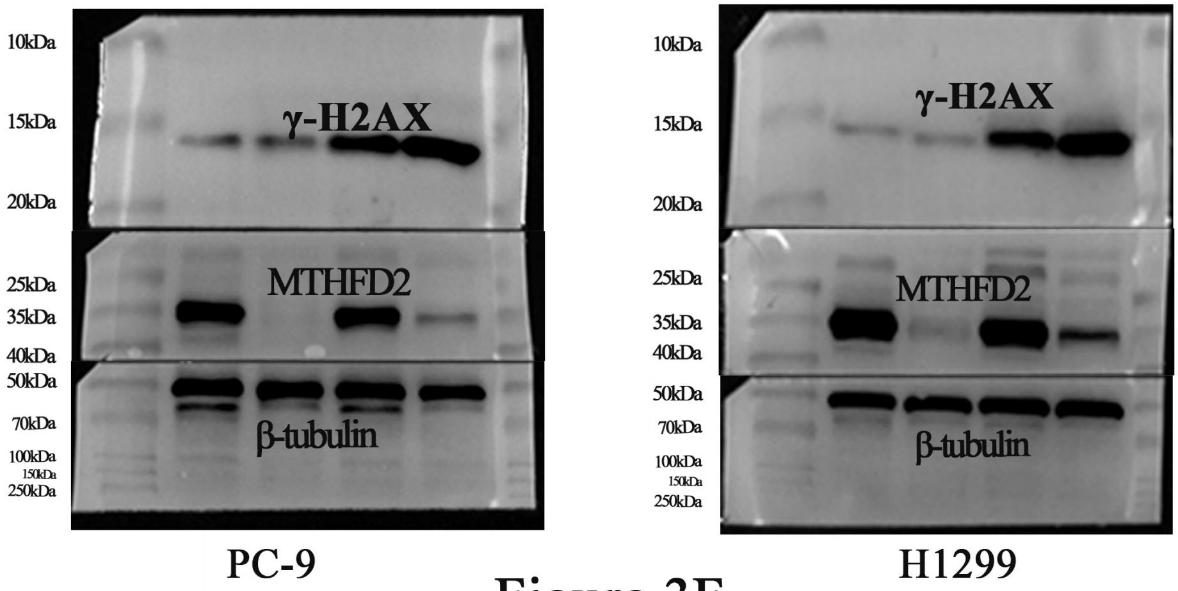
10kDa
15kDa
20kDa
25kDa
35kDa
40kDa
50kDa
70kDa
100kDa
150kDa
250kDa
γ-H2AX
MTHFD2
β-tubulin
PC-9
H1299

Figure 3F

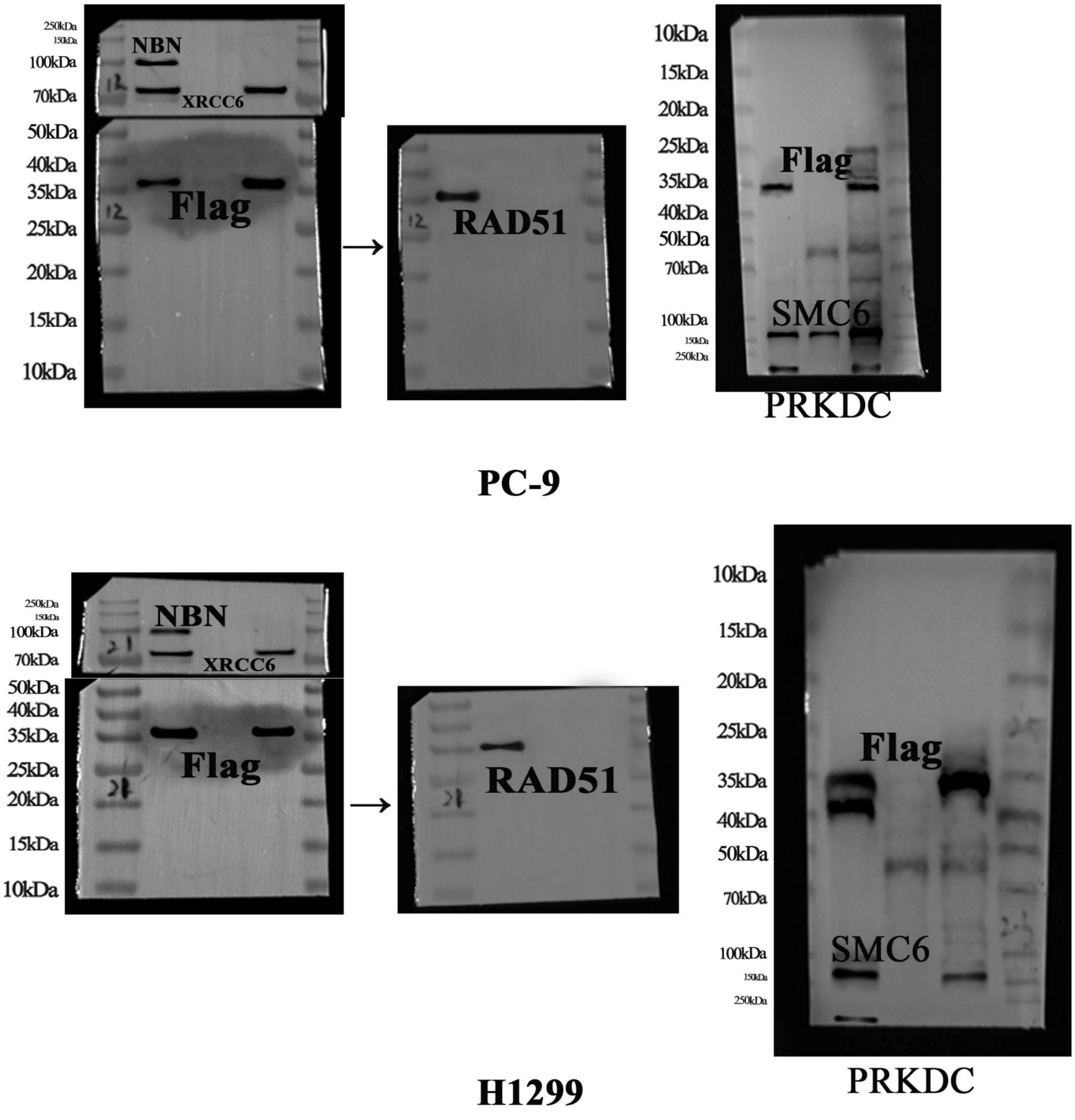
250kDa
150kDa
100kDa
70kDa
50kDa
40kDa
35kDa
25kDa
20kDa
15kDa
10kDa
NBN
XRCC6
Flag
RAD51
SMC6
PRKDC
PC-9
H1299

Figure 4B

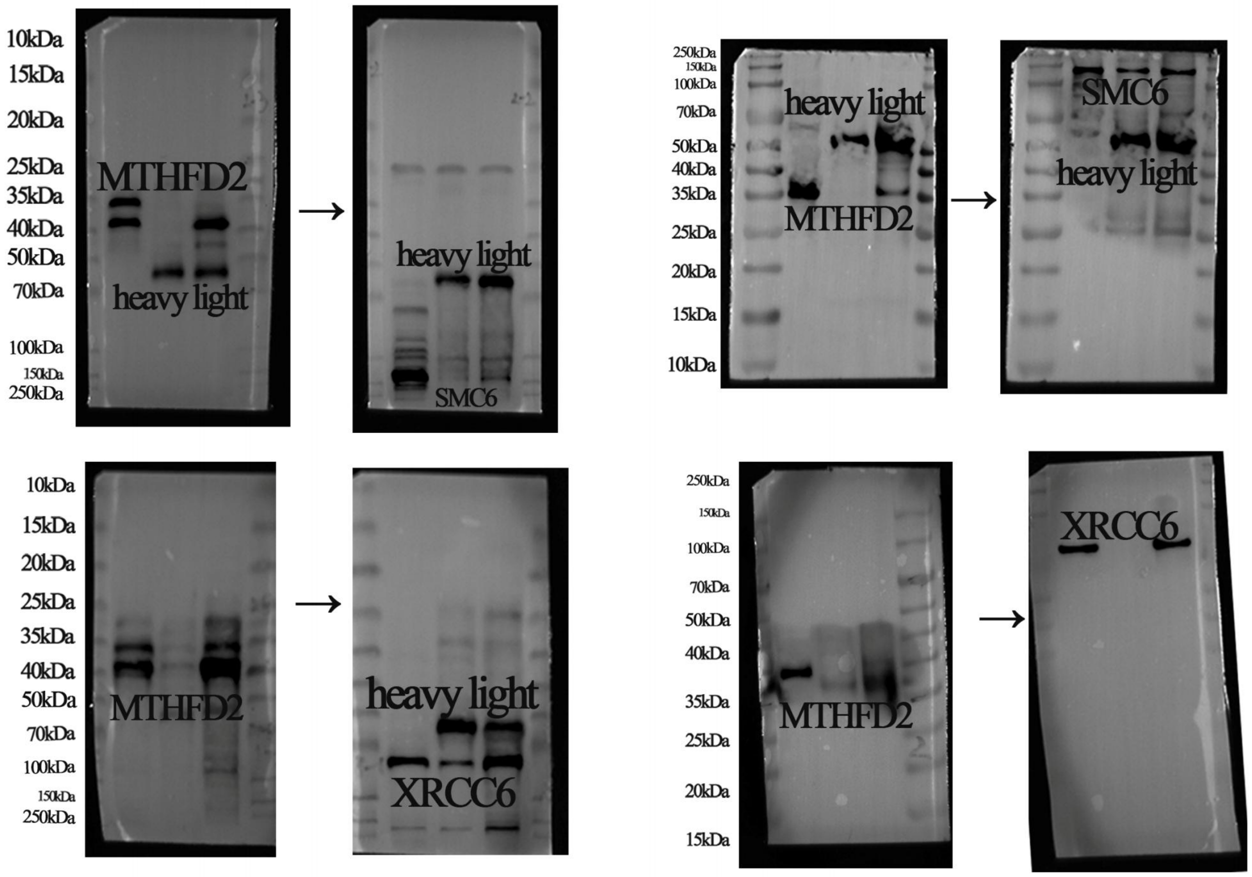

PC-9 H1299

Figure 4C

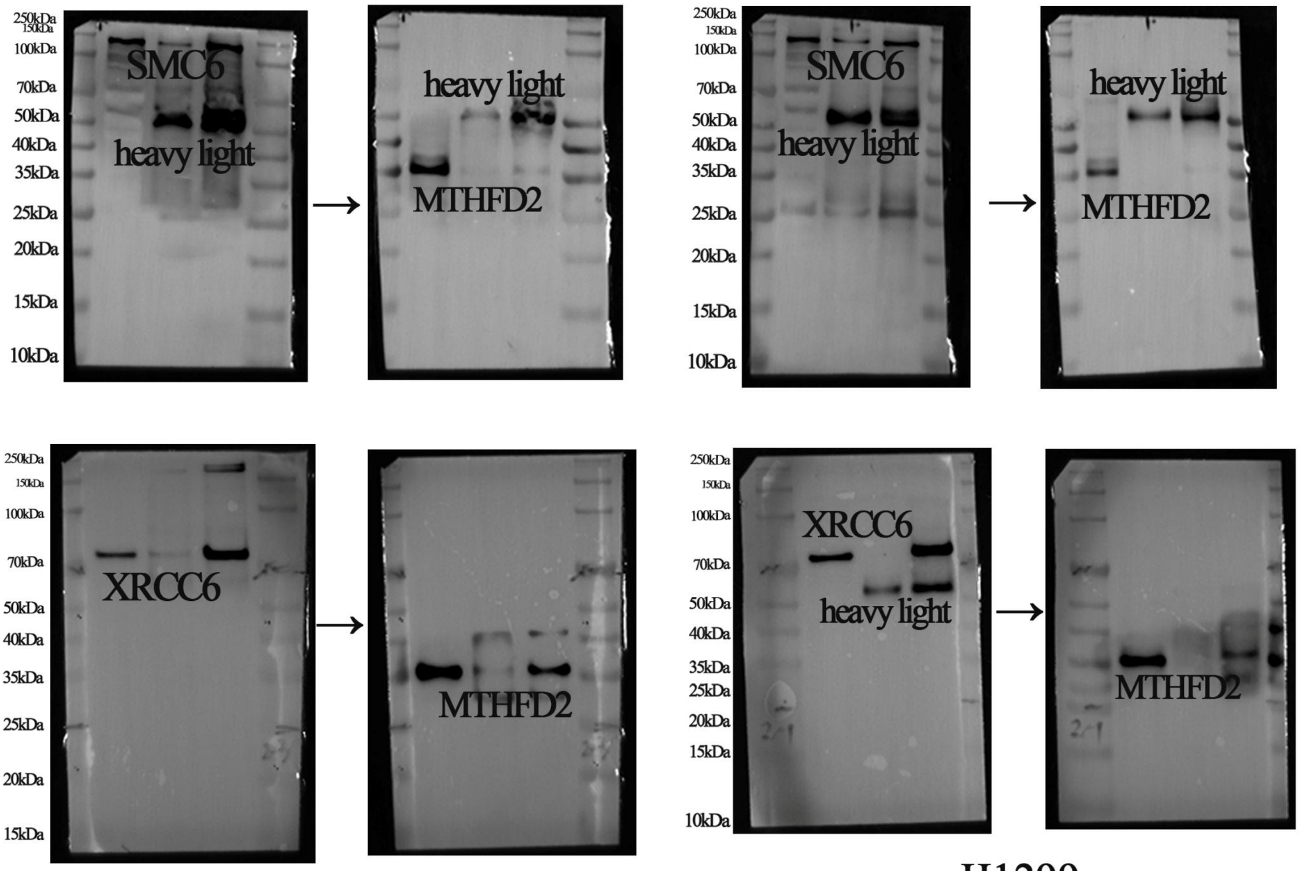

PC-9

H1299

Figure 4D

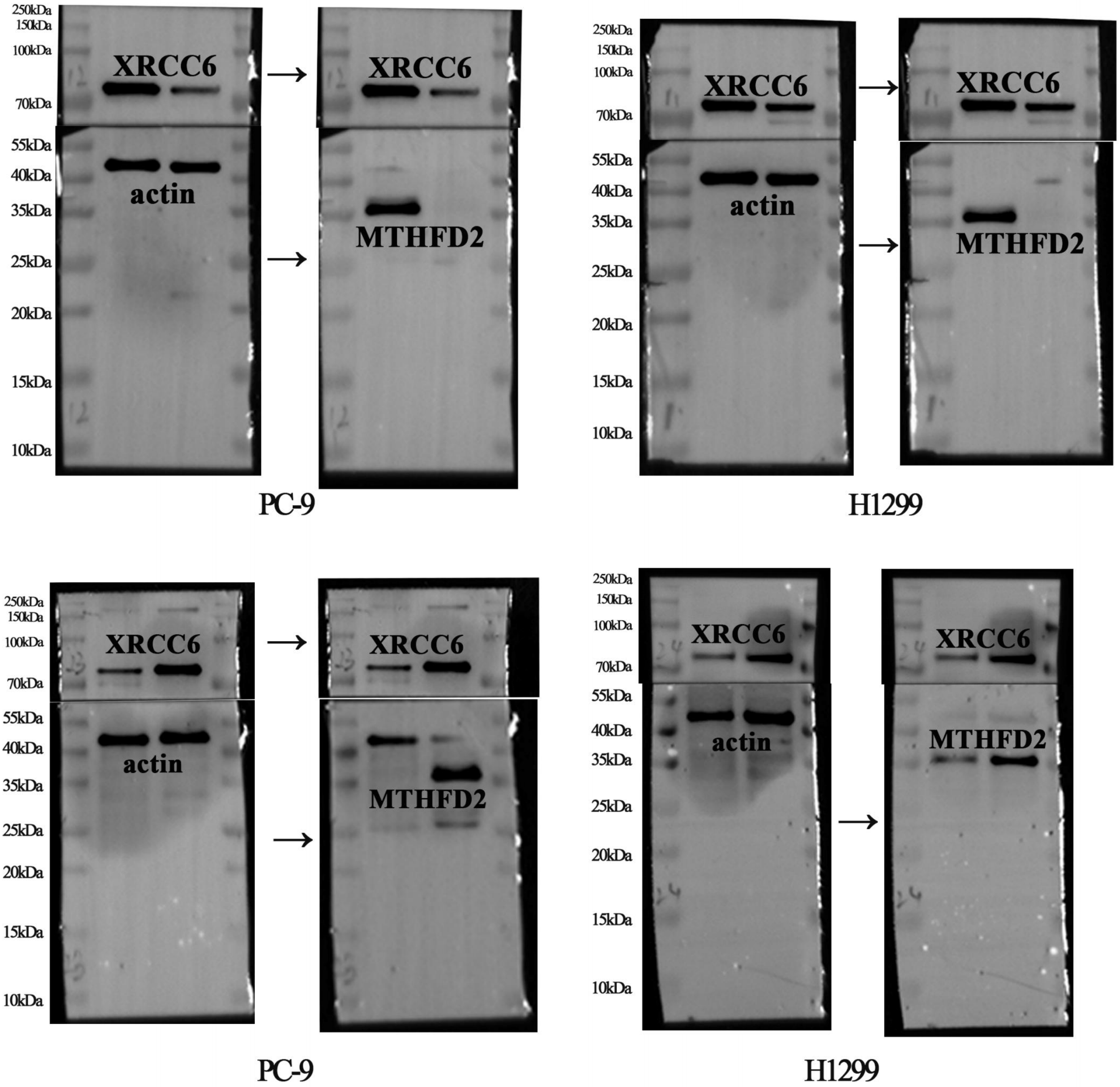
250kDa
150kDa
100kDa
70kDa
55kDa
40kDa
35kDa
25kDa
20kDa
15kDa
10kDa
XRCC6
actin
MTHFD2
PC-9
H1299

Figure 4F

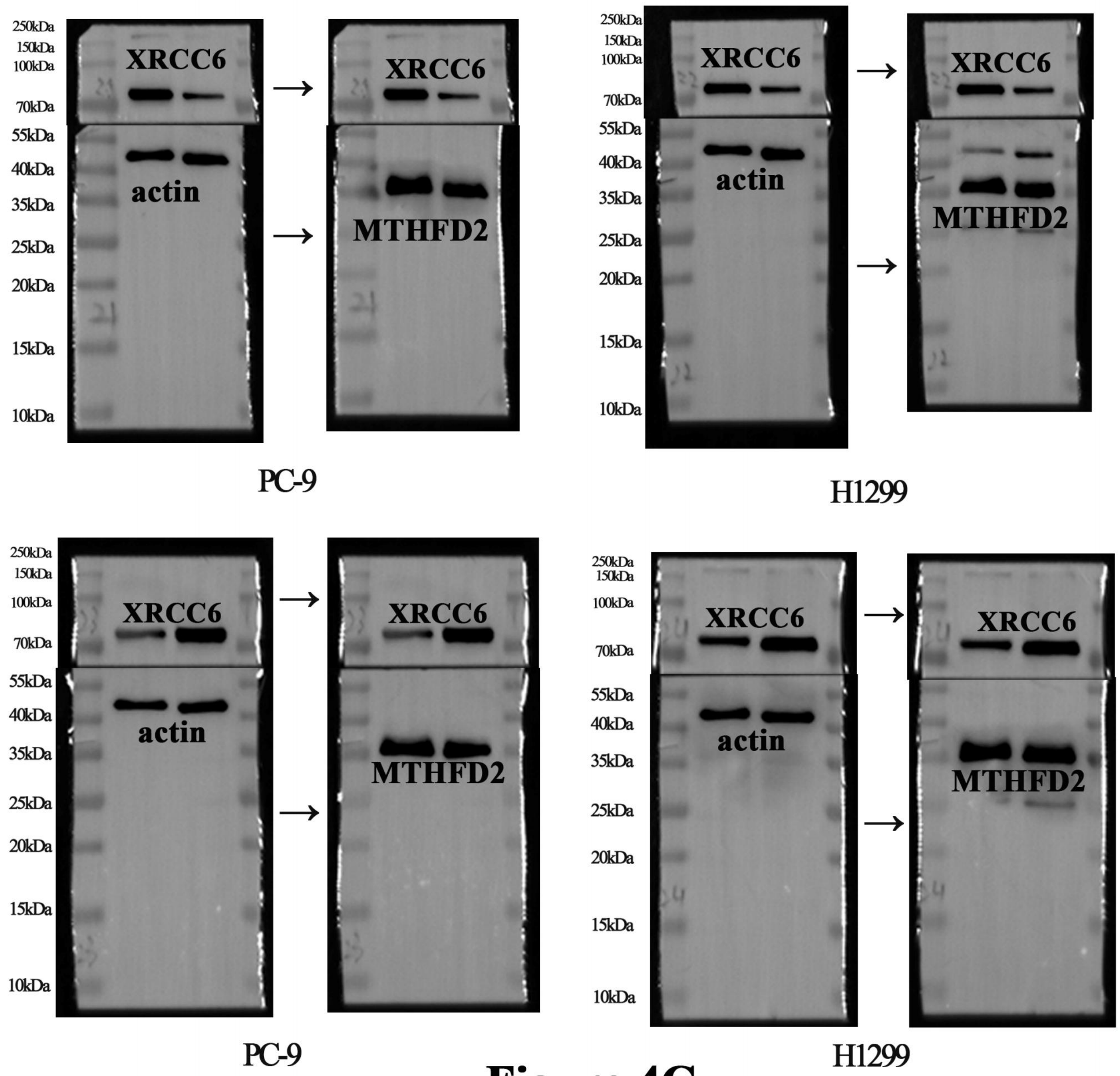
250kDa
150kDa
100kDa
70kDa
55kDa
40kDa
35kDa
25kDa
20kDa
15kDa
10kDa
XRCC6
actin
MTHFD2
PC-9
H1299

Figure 4G

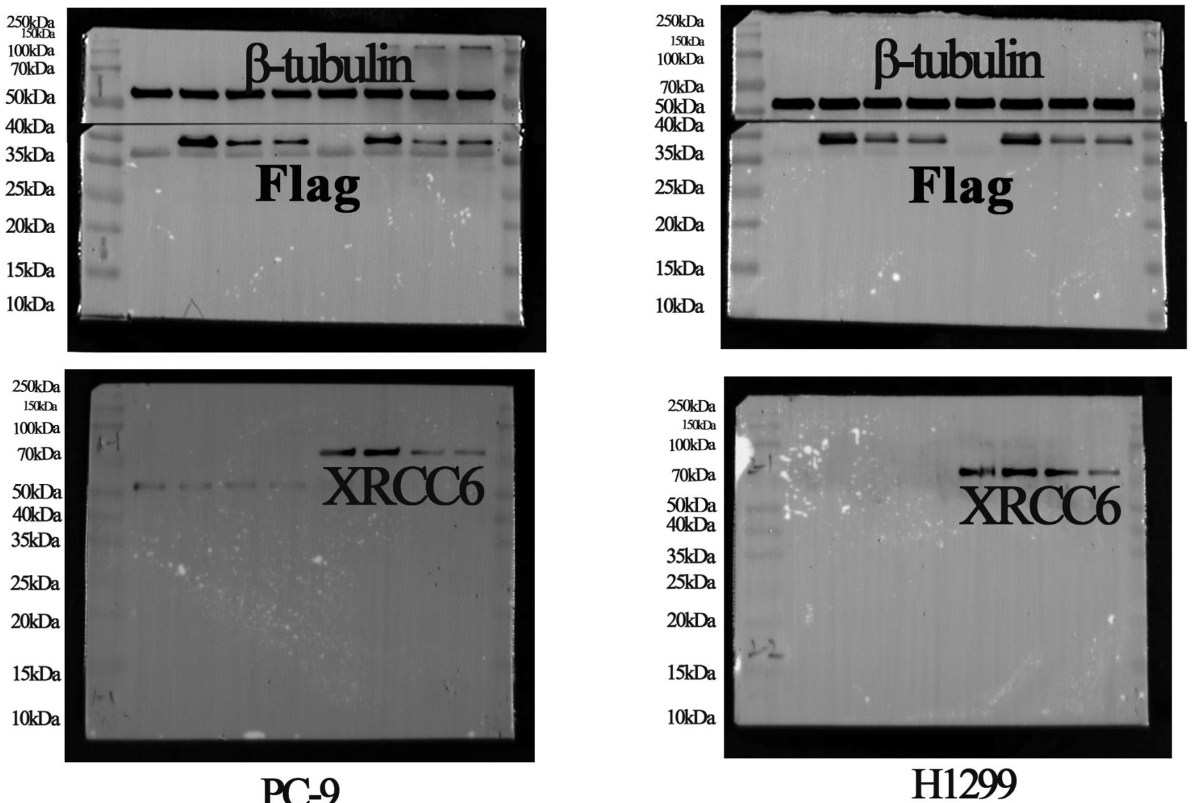
250kDa
150kDa
100kDa
70kDa
50kDa
40kDa
35kDa
25kDa
20kDa
15kDa
10kDa
β-tubulin
Flag
XRCC6
PC-9
H1299

Figure 4H

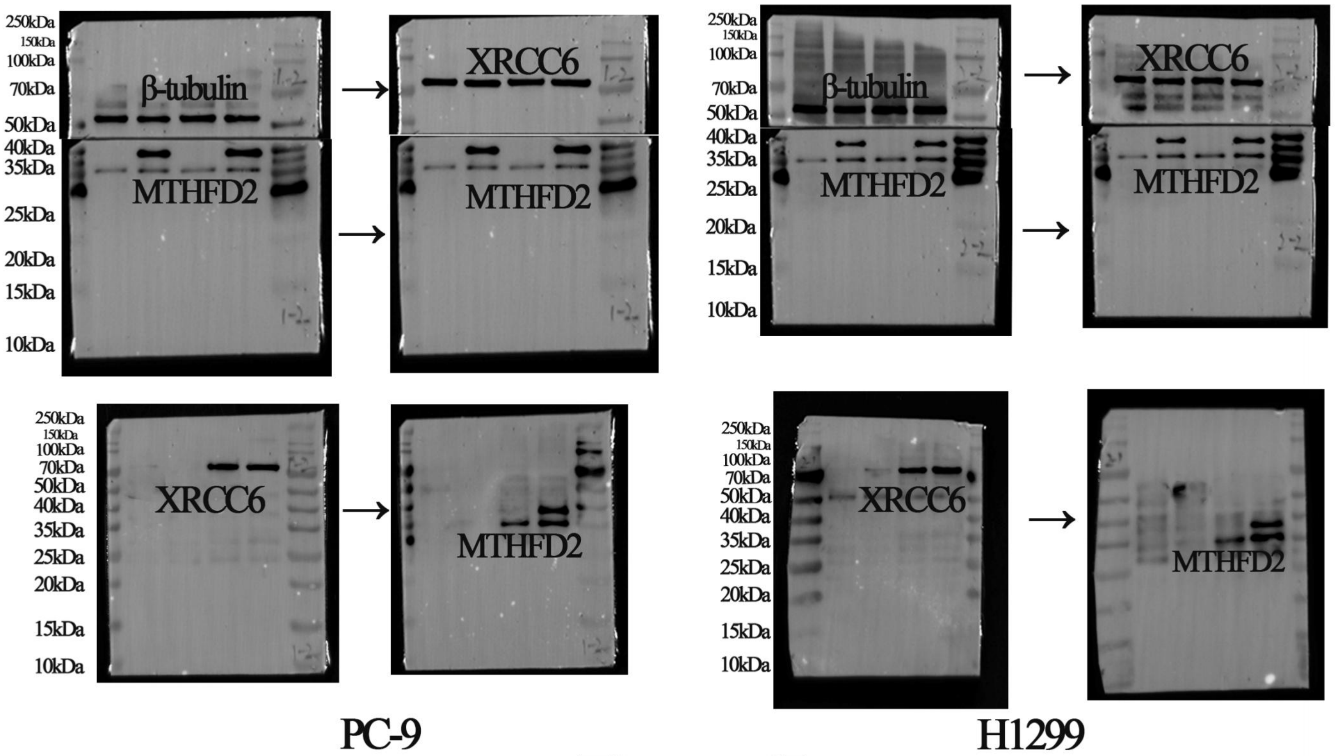
250kDa
150kDa
100kDa
70kDa
50kDa
40kDa
35kDa
25kDa
20kDa
15kDa
10kDa
β-tubulin
XRCC6
MTHFD2
XRCC6
MTHFD2
PC-9
H1299

Figure 4I

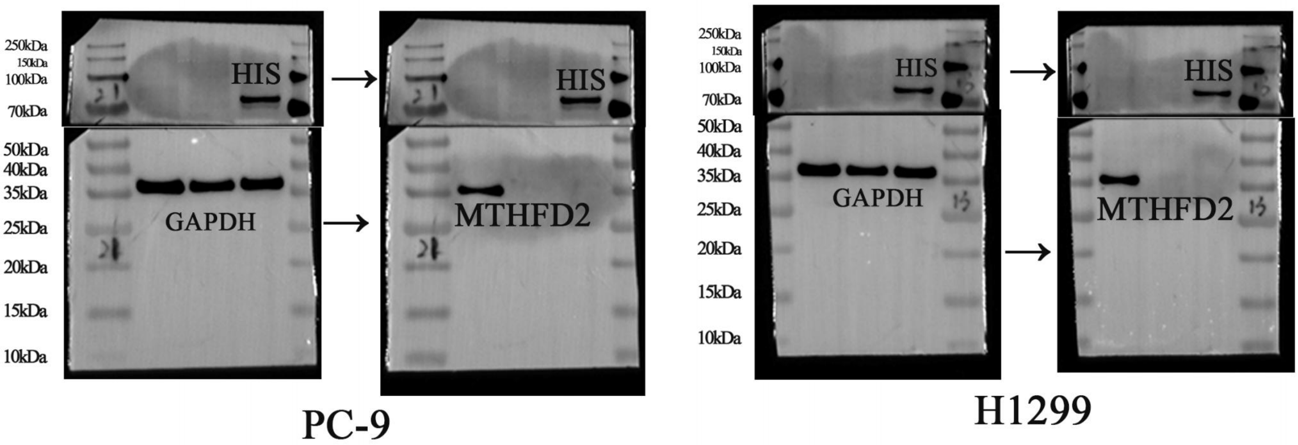

250kDa
150kDa
100kDa
70kDa
50kDa
40kDa
35kDa
25kDa
20kDa
15kDa
10kDa
HIS
GAPDH
MTHFD2
PC-9
H1299

Figure 6A

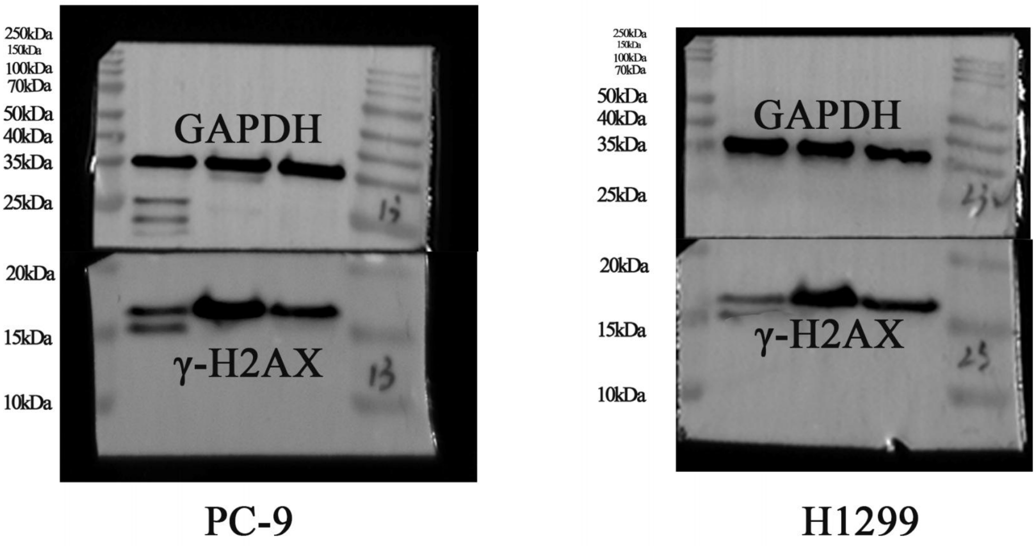
250kDa
150kDa
100kDa
70kDa
50kDa
40kDa
35kDa
25kDa
20kDa
15kDa
10kDa
GAPDH
γ-H2AX
PC-9
250kDa
150kDa
100kDa
70kDa
50kDa
40kDa
35kDa
25kDa
20kDa
15kDa
10kDa
GAPDH
γ-H2AX
H1299

Figure 6D

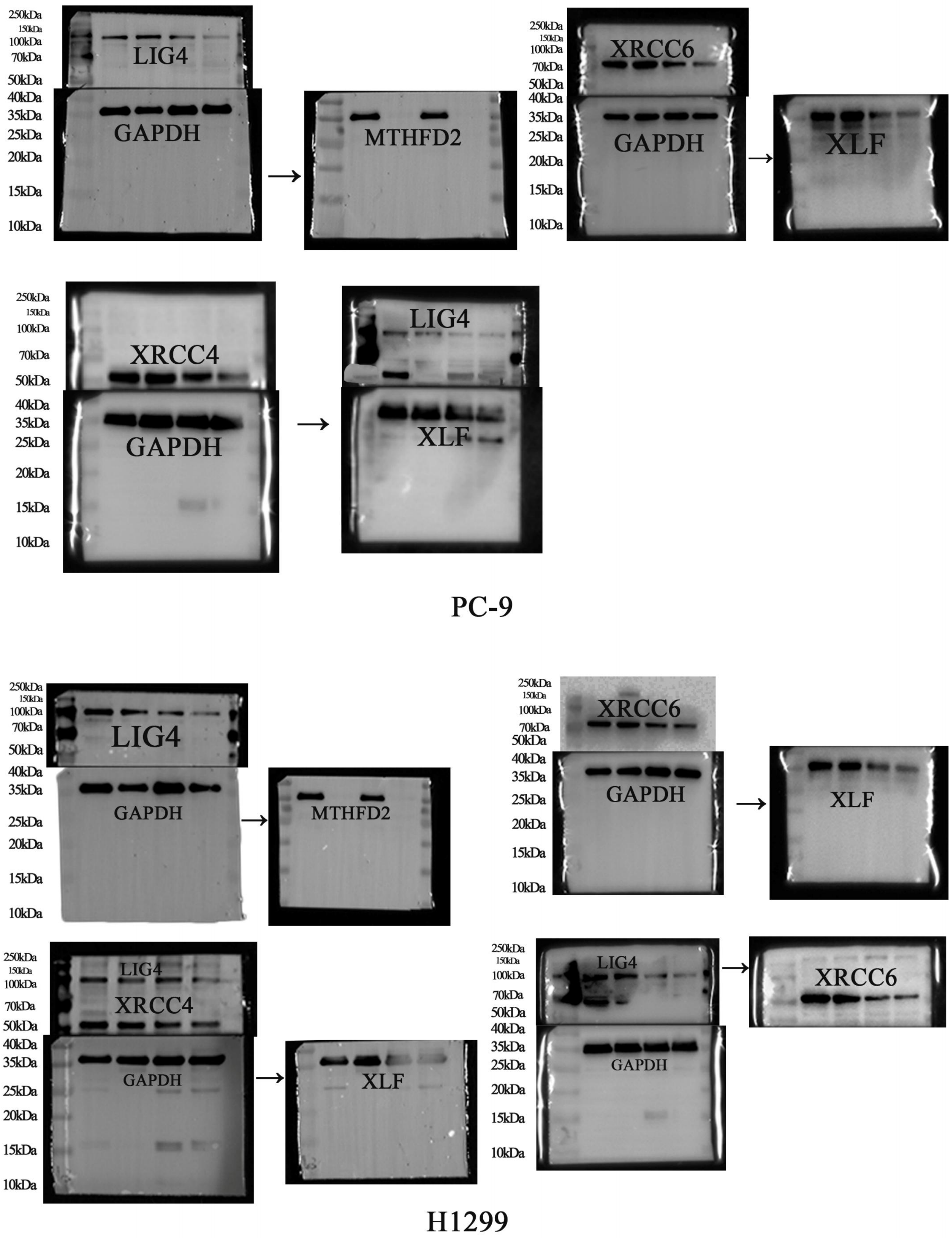
LIG4
GAPDH
MTHFD2
XRCC6
XLF
XRCC4
250kDa
150kDa
100kDa
70kDa
50kDa
40kDa
35kDa
25kDa
20kDa
15kDa
10kDa
PC-9
H1299

Figure 7A
